# Unraveling the effect of intra- and intercellular processes on acetaminophen-induced liver injury

**DOI:** 10.1038/s41540-022-00238-5

**Published:** 2022-08-06

**Authors:** M. M. Heldring, A. H. Shaw, J. B. Beltman

**Affiliations:** Division of Drug Discovery and Safety, Leiden Academic Centre for Drug Research, Leiden University, Einsteinweg 55, 2333 CC Leiden, The Netherlands

**Keywords:** Virtual drug screening, Dynamical systems, Computer modelling

## Abstract

In high dosages, acetaminophen (APAP) can cause severe liver damage, but susceptibility to liver failure varies across individuals and is influenced by factors such as health status. Because APAP-induced liver injury and recovery is regulated by an intricate system of intra- and extracellular molecular signaling, we here aim to quantify the importance of specific modules in determining the outcome after an APAP insult and of potential targets for therapies that mitigate adversity. For this purpose, we integrated hepatocellular acetaminophen metabolism, DNA damage response induction and cell fate into a multiscale mechanistic liver lobule model which involves various cell types, such as hepatocytes, residential Kupffer cells and macrophages. Our model simulations show that zonal differences in metabolism and detoxification efficiency are essential determinants of necrotic damage. Moreover, the extent of senescence, which is regulated by intracellular processes and triggered by extracellular signaling, influences the potential to recover. In silico therapies at early and late time points after APAP insult indicated that prevention of necrotic damage is most beneficial for recovery, whereas interference with regulation of senescence promotes regeneration in a less pronounced way.

## Introduction

Acetaminophen (paracetamol, APAP) is safe to use at therapeutic doses, but can cause acute liver injury (ALI) when overdosed. Susceptibility to APAP-induced liver failure is variable among individuals, which for example is apparent from differences in responses between groups of different sex^1–3^, age^4–8^ and health status^9–13^. To gain insight into factors that sensitize the liver to ALI and therefore could be therapeutic targets, a quantitative understanding of APAP metabolism, subsequent toxicity, hepatic injury and recovery is needed. These processes do not only depend on intracellular dynamics of hepatocytes, but are in part regulated by intercellular signaling between hepatocytes and immune cells such as the liver’s resident Kupffer cells (KCs) and recruited monocyte-derived macrophages (mϕs)^14^. Therefore, we need to consider spatial organization and its effect on intracellular processes simultaneously.

Liver tissue is structured in hepatic lobules. APAP in the blood plasma enters the liver via hepatic portal veins (PVs), where it flows through sinusoids in the liver lobule in the direction of the central vein (CV). Lining the sinusoids are hepatocytes that take up about 95–98% of APAP, whereas about 2–5% of the drug leaves the body via urine in unchanged form^15–17^. Approximately 90% of the intracellular APAP is metabolized by glucuronidation and sulfation pathways, whereas 5–10% is oxidized by cytochrome P450 (P450) enzymes to form the toxic metabolite NAPQI^18,19^. NAPQI is detoxified and subsequently excreted by glutathione (GSH). However, high NAPQI concentrations lead to GSH depletion, which enables covalent binding of NAPQI to cysteine residues (NAPQI-cys) on proteins^20^. Interestingly, cytochrome P450 and GSH are not equally distributed across the lobule, due to liver lobule zonation^21,22^. Hepatocytes closest to the central vein have high levels of cytochrome P450 and relatively low numbers of GSH molecules^23,24^. Therefore, hepatocytes in the centrilobular area produce more NAPQI and deplete GSH faster than cells in the periportal area^25,26^. As a result, those cells accumulate more NAPQI-cys which induces damage primarily in the pericentral area.

NAPQI-cys adducts cause oxidative stress, c-Jun N-terminal kinase (JNK) activation and amplification of mitochondrial oxidative stress that can result in mitochondrial membrane permeability transition pore opening, an inability to synthesize ATP and ultimately necrosis^27–30^. In addition, mitochondrial dysfunction can lead to the release of endonuclease G that causes nuclear DNA fragmentation which contributes to cell death^27,29^, but has also been associated to senescence^31,32^. Mild APAP-induced stress and consequent nuclear DNA damage can also activate the DNA damage response (DDR) pathway^33–35^, cell cycle arrest^36^, and can trigger senescence^14,35^. The DDR regulatory protein p53 transcriptionally activates p21 and its own inhibitor MDM2^37–39^. The protein p21 is a cell cycle inhibitor and can induce either temporary cell cycle arrest or senescence^40^. APAP-induced senescence is most prevalent among cells at the perinecrotic area, where NAPQI-cys levels are high. Senescent cells typically secrete an inflammatory mix of signals, known as senescence-associated secretory phenotype (SASP)^41^. One important component of SASP is transforming growth factor beta (TGF-β). This cytokine can modulate the expression of downstream target genes, among which is p21, through activation of transcription factors such as SMAD3^42^. Therefore, in addition to cell-autonomous senescence induced via p53, p53-independent senescence can be triggered by extracellular TGF-β^43^. This implies that neighboring hepatocytes may become senescent due to exposure of paracrine SASP signals from senescent cells in close proximity^14^.

Because APAP-induced liver injury typically manifests with pericentral necrosis surrounded by senescent hepatocytes, the damage does not occur equally throughout liver lobules, but the damaged site is surrounded by undamaged hepatocytes. Clearance of the necrotic site and regeneration through proliferation of healthy cells are necessary for complete recovery after injury, yet such recovery is likely hampered by senescent cells that can no longer proliferate. For both clearance and proliferation, macrophages that are recruited to the damaged area play an important role. This recruitment occurs because Kupffer cells excrete monocyte chemoattractant protein 1 (MCP-1) in response to damage associated molecular patterns (DAMPs) released by necrotic hepatocytes^44,45^. In addition to the removal of necrotic debris^46–50^, the recruited macrophages excrete TGF-β through which they stimulate senescence^14^ and mitogens such as interleukin 6 (IL-6) and tumor necrosis factor alpha (TNF-α) that trigger proliferation^51–54^. Further spread of senescence due to macrophage-derived TGF-β signaling can therefore counteract the pro-regenerative effect of mitogens. Thus, these contrasting processes should be tightly regulated to prevent a disbalance favoring one of the two cell fate outcomes.

There are numerous studies that describe the various processes involved in APAP-induced liver injury using mathematical models. Among the most well-known and complete is the commercial DILIsym® software, that can be employed to predict potential drug hazards, such as the carcinogenic potential of APAP^55^, or to elucidate mechanisms of toxicity, e.g. for the APAP and its isomer 3'-Hydroxyacetanilide (AMAP)^56^. Other research has modeled subcomponents of APAP-induced hepatotoxicity. For example, APAP metabolism models that incorporate detoxification by sulfation and glucuronidation as well as oxidation and GSH conjugation reactions in ordinary differential equations (ODEs) can be used to study parameter sensitivity and to generate virtual populations^57–60^. Some of these models include molecular gradients to account for liver zonation and the concomitant spatial differences in toxicity^59,60^. Other studies have extended the ODEs describing APAP metabolism with predictions on hepatocyte damage and cell death markers^61,62^, for example to estimate the amount of overdose, time since overdosing and outcome for patients^61^. Other computational models have focused explicitly on the spatial organization of the liver and use that to describe distribution of xenobiotics^59,63^, regeneration^64^, or APAP-induced damage and recovery^65^. Moreover, to improve our understanding and aid our interpretation of biomarker release after hepatic injury, a quantitative model has been developed to describe the spatiotemporal mechanisms that cause APAP-induced excretion of serum alanine aminotransferase (ALT) levels and hepatic necrosis^66,67^.

However, none of these published models integrates spatial organization of hepatocytes and metabolic zonation with senescence, necrosis and regeneration, taking the influence of immunosurveillance by KCs and mϕs on outcome into account. Therefore, we built a comprehensive spatial model of a human liver lobule that incorporates an intracellular APAP metabolism model^58^, our previously constructed model for the activation of the DDR^68^, hepatocyte damage, and interaction with KCs and mϕs. We used this model to study the regenerative capacity of the system by manipulation of zonation and perturbation of parameters, thereby demonstrating that senescence and proliferation events are expected to be influenced by alterations in intracellular metabolic and stress signaling dynamics, in addition to extracellular stimulants. We subsequently used our model to simulate and predict the effect of various therapies on liver damage and a liver’s potential to recover from APAP-induced injury.

## Results

### A spatiotemporal model accurately describes APAP metabolism within a liver lobule

**Fig. 1:**
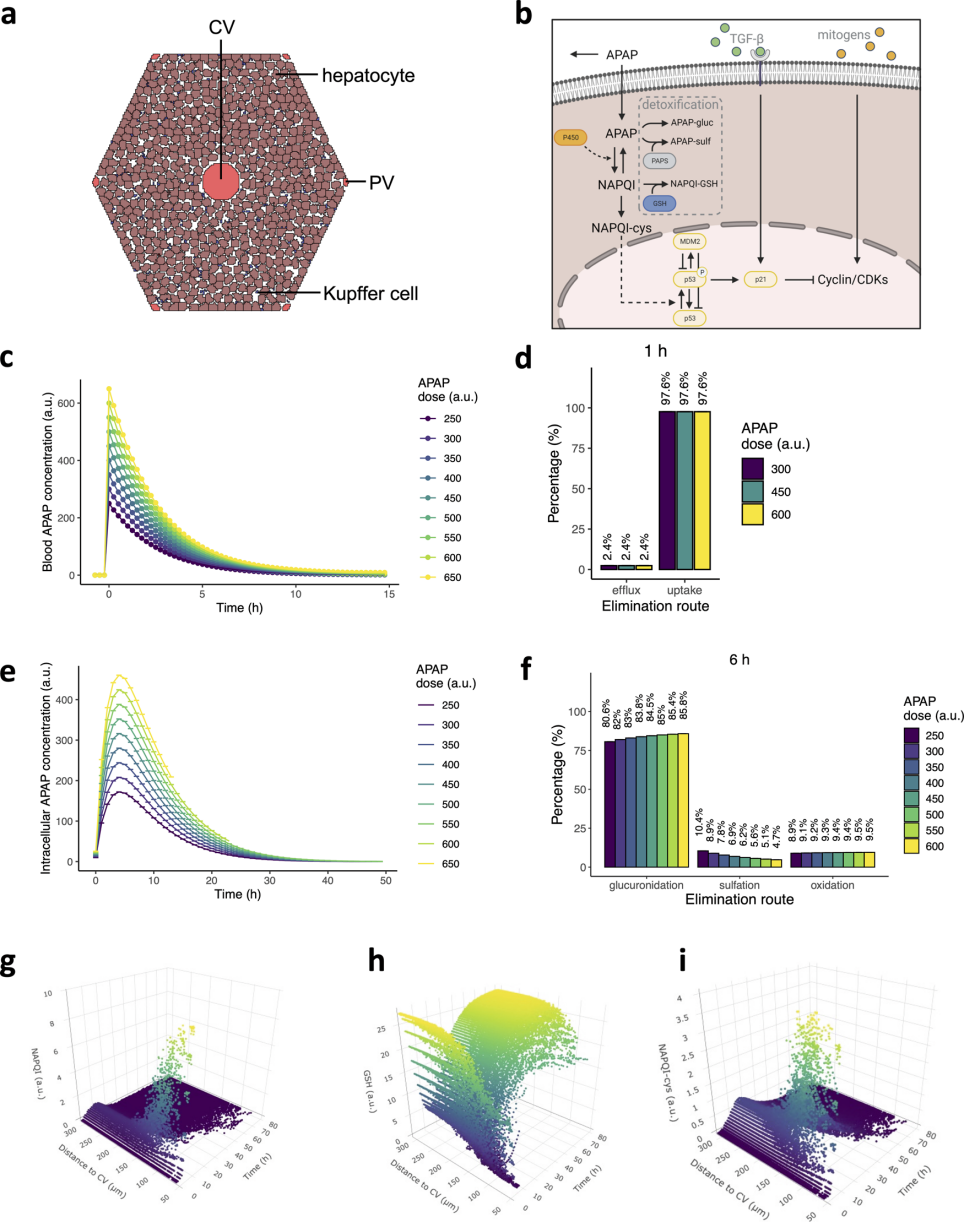
In silico simulations of one liver lobule that accurately describe APAP metabolism in hepatocytes. **a** Initial state of the spatial model structure of one lobule, with the central vein (CV) and six portal veins (PVs). The brown cells are hepatocytes, and the small blue cells represent Kupffer cells (KCs). Empty, white space represents other non-parenchymal cells, such as sinusoidal epithelial cells and stellate cells, and intercellular spaces. **b** Representation of modeled APAP metabolism, DNA damage response activation mediated by p53, and regulation of cyclin/cyclin-dependent kinases (CDKs) through p21 and mitogenic signaling. Note that we did not explicitly include all components involved in the modeled interactions; for example, we omitted mitochondrial damage inflicted by NAPQI-cys and consequent release of endonucleases, leading to DNA fragmentation. Figure created with BioRender. **c** APAP concentration in the blood plasma over time for different starting concentrations. **d** Percentage of APAP that is excreted as unmodified compound or taken up by hepatocytes at *t* = 1 h after exposure. **e** Hepatocellular APAP concentration over time for different initial APAP concentrations. **f** Percentages of APAP in each elimination route at 6 h after exposure. **g–i** NAPQI (**g**), GSH (**h**) and NAPQI-cys (**i**) concentration as function of distance from the central vein and time after exposure for an initial APAP concentration of 450. Pericentral necrotic cell death and recovery is noticeable from the absence and reappearance of data points in the area with low distance from the CV. Note that simulations were performed at different starting APAP concentrations with ten repeats per condition (represented as mean ± sd), yet in **c**–**f** there is no variability between simulations per condition due to the deterministic character of the intracellular dynamics. a.u., arbitrary units.

To study the sensitivity of the liver to variations in the molecular and systemic processes after APAP exposure, we created a spatiotemporal model of one human liver lobule that describes hepatocellular APAP uptake and metabolism, and the effect of these intracellular dynamics on initiation, progression and regression of injury. To this purpose, we used a 2D cellular Potts model (CPM), which is a grid-based model formalism taking into account cellular shape and motility by means of a Hamiltonian^69,70^. The model simulates the two-dimensional hexagonal contours of a liver lobule, with ‘fixed’ portal veins in the corners and a central vein in the center of the lobule, and hepatocytes and Kupffer cells in the lobular area (Fig. 1a). In addition, we used separate scalar fields to model the diffusion of extracellular APAP, chemokines and cytokines across the lobule. Hepatocellular APAP uptake and metabolism (Fig. 1b), and subsequent activation of the DNA damage response were established by modifying existing models (Supplementary Fig. 1)^58,68^ and applying them to individual hepatocytes, while accounting for the zonal differences in GSH and cytochrome P450 concentrations.

We used the resulting model to simulate the effect of different blood concentrations of APAP on the intracellular metabolism and DDR pathway activation in human hepatocytes. From studies that monitored APAP blood plasma concentrations in humans and mice, we deducted that the ratio between the administered dose (oral for human and intraperitoneal for mice, in mg/kg) and the plasma concentration within the first 15–30 min after exposure (in µg/ml) is ~1 (Supplementary Table 1)^19,25,71–75^. Therefore, we chose a range of initial APAP values between 250 for low and 650 for high levels of adversity that match realistic levels of APAP in the blood plasma^76^ and that correspond to similar administration quantities (albeit expressed in mg/kg). Note that we decided to use arbitrary units (a.u.) instead of mg/kg as unit throughout our model simulations, because the concentration units for the DDR model are not known. Combining these models with explicit interpretation of concentration units would thus also imply defined concentration units for the DDR model that we currently cannot verify. The parameters for APAP uptake and excretion were chosen such that APAP levels in the blood decreased to very low levels within 12 h after exposure (Fig. 1c), corresponding to a half-life ${{{\mathrm{t}}}}_{\frac{1}{2}} \approx 2$ h as previously reported^18,77–80^. The utilized parameterization for APAP elimination resulted in ~2.4% direct APAP excretion and 97.6% of uptake by hepatocytes, irrespective of exposure dosage (Fig. 1d), i.e., similar to reported values^15–17^. The intracellular APAP concentration peaked shortly after exposure and rapidly decreased due to glucuronidation, sulfation and oxidation (Fig. 1e). Glucuronidation and sulfation accounted for ~80–85% and 5–10% of the APAP detoxification, respectively (Fig. 1f), which roughly corresponds to experimental studies in humans and rats^19,81,82^. The remaining 10% of APAP was oxidized by cytochrome P450 into NAPQI, with only a limited dependence on the initial APAP concentration (Fig. 1f). Due to liver zonation, the intracellular NAPQI (Fig. 1g), GSH (Fig. 1h) and NAPQI-cys (Fig. 1i) concentrations differed greatly among cells, with the highest NAPQI-cys concentrations in the perivenous region. Thus, our model accurately describes APAP metabolism within a liver lobule, while taking its zonation into account.

### Model simulations predict a threshold APAP concentration above which liver recovery is not possible

**Fig. 2:**
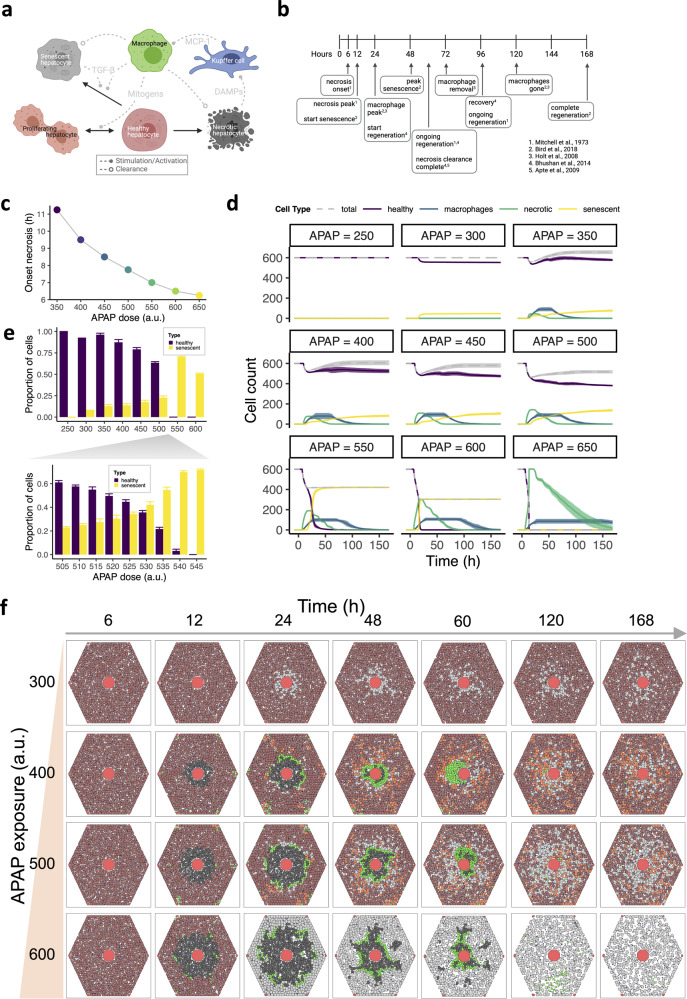
In silico simulations match experimental observations of damage and recovery and predict a threshold concentration beyond which recovery does not occur. **a** Representation of hepatic cell state transitions and intercellular signaling in our model. Healthy hepatocytes can proliferate, become senescent or necrotic. Necrotic hepatocytes activate resident Kupffer cells (KCs) through production of DAMPs. KCs excrete MCP-1 to recruit macrophages that produce TGF-β and mitogens to stimulate senescence or proliferation of healthy hepatocytes, respectively. Figure created with BioRender. **b** Reference timeline for key processes in liver tissue following exposure to an intermediate APAP concentration (~400 mg/kg), based on indicated literature. Figure created with BioRender. **c** Timing of necrosis onset, defined as the time point at which the first necrotic hepatocytes appeared, after exposure to different APAP concentrations. Due to the deterministic character of the intracellular dynamics, there is no variation between different simulations per condition. **d** Counts of healthy (purple), necrotic (green) and senescent (yellow) hepatocytes and macrophages (blue) over time after exposure to different APAP concentrations. The gray dashed line represents the total number of alive hepatocytes, i.e., the sum of healthy and senescent hepatocytes. **e** Proportion of healthy or senescent cells and the total of healthy and senescent cells, at day 7 after exposure to different APAP concentrations. **f** Stills of model simulation at different time points in hours (columns) and initial APAP concentrations (rows). Brown, healthy hepatocytes; red, CV and PVs; black, necrotic hepatocytes; gray, senescent hepatocytes; green, macrophages; orange, proliferated hepatocytes; blue, Kupffer cells. Results are based on 10 simulations per condition represented as mean ± sd. a.u., arbitrary units.

In addition to simulations of intracellular biochemical reactions, we utilized the model to simulate tissue damage and regeneration influenced by extracellular signaling, considering necrotic, senescent, and proliferating hepatocytes (Fig. 2a). We set model parameters such that the various phases of damage and recovery took place during approximately realistic time periods (Fig. 2b). In silico hepatocytes that accumulated very high levels of NAPQI-cys became necrotic between 6.25 and 11.25 h after APAP administration (Fig. 2c), similar to necrosis onset at 6 h measured in mice^25^ and predictions in humans^61,83^. We modeled expression of DAMPs by necrotic cells, thereby activating neighboring Kupffer cells, which in turn initiated MCP-1 excretion to recruit monocyte-derived macrophages originating from the periportal region and blood. In silico macrophages were responsible for clearance of senescent and necrotic hepatocytes. In our model simulations, the number of macrophages increased rapidly within 24 h after injury (Fig. 2d), which corresponded to observations in mice^14,47,84^.

Hepatocytes with high intracellular NAPQI-cys concentrations that did not exceed the threshold for necrosis were considered to become senescent via p53 activation followed by elevated p21 expression (Fig. 2d). In addition to this cell-autonomous senescence, in silico senescent hepatocytes as well as macrophages excreted TGF-β that further stimulated p21 production in a p53-independent manner, thereby enhancing the occurrence of senescence in surrounding hepatocytes. Opposing pro-senescent signaling, we also included macrophage-mediated stimulation of proliferation by healthy hepatocytes in our model, which was essential for successful regeneration of damaged liver tissue.

Exposure to a low APAP blood concentration did not induce necrotic damage, whereas several hepatocytes became senescent in the perivenous region after 24 h (Fig. 2d, e; top row in Fig. 2f). Intermediate APAP concentrations, i.e., from ~350 to ~500 led to mild to severe liver injury characterized by early occurrence of necrosis and subsequent senescence, necrotic clearance between 60 and 72 h, depending on the severity of injury, and regeneration (Fig. 2d, e; middle rows in Fig. 2f; Supplementary Movie 1). Full recovery of the lobule in the high dose conditions, i.e., 550 or higher, was not possible due to the transition of all healthy hepatocytes into a necrotic or senescent state (Fig. 2d; bottom row in Fig. 2f). Because a sharp transition from some to all cells becoming senescent occurred between 500 and 550 APAP (Fig. 2e), we ran additional simulations for APAP doses in between these values (Supplementary Fig. 2a). The decrease in the proportion of healthy cells dropped rapidly at doses >530, which indicates there is a tipping point at which too few healthy cells remain to ensure sufficient recovery (Fig. 2e).

To examine the effect of changes in parameters that directly impact cell fate on the model outcome, we decreased the probability of necrosis (p_necrosis_) and varied the degradation rates of DAMPs (d_D_) and MCP-1 (d_M_). Changes in p_necrosis_ had little effect on the outcome (Supplementary Fig. 2b), whereas a hundred-fold decrease in degradation rates for DAMPs and MCP-1 caused a rapid increase in the number of macrophages and consequent proliferation (Supplementary Fig. 2c, d). This high number of macrophages had little impact on the transmission of senescence, because only a small proportion of macrophages was in direct contact with necrotic cells and produced TGF-β, which caused a high abundance of mitogens compared to TGF-β. Overall, our model accurately simulated the dose-dependency of adversity and recovery patterns of liver tissue after APAP administration and predicts a threshold APAP concentration above which an ‘average’ liver cannot recover.

### Absence of GSH and P450 gradients increase the APAP sensitivity

Because the susceptibility to develop adversity and the severity of adversity can vary extensively between individuals, we sought to understand the sensitivity of the outcome to variations in the underlying processes. To this end, we distinguished the metabolic pathway, the DNA damage response and cell fate modulation by macrophages as the three main regulatory pathways that might influence the extent of interindividual variability.

**Fig. 3:**
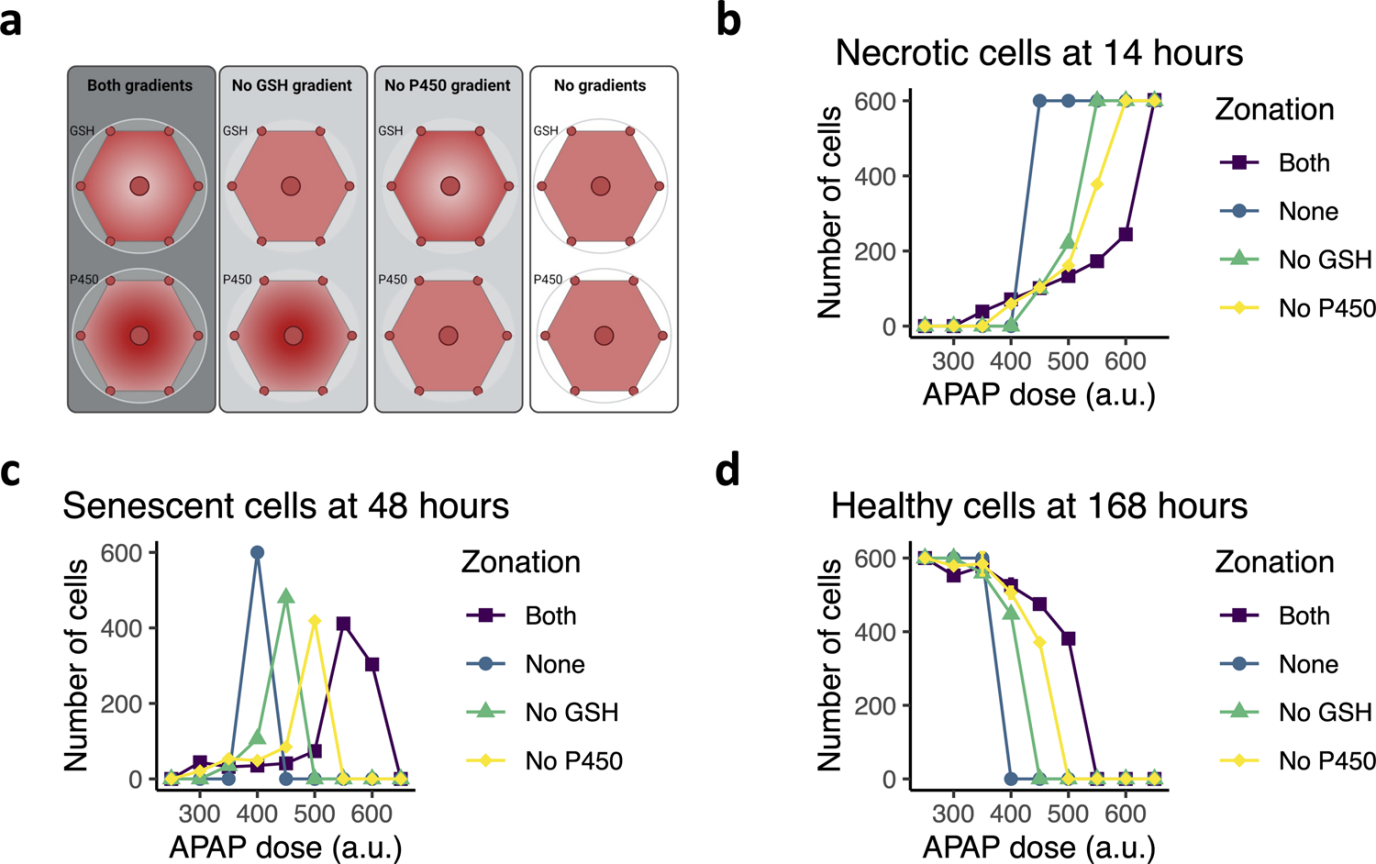
The effect of zonal expression of GSH and P450 on adverse outcome. **a** Illustration of four different in silico zonation scenarios. Figure created with BioRender. **b**–**d** The dependence of the number of necrotic hepatocytes at 14 h after exposure (**b**), of the number of senescent hepatocytes at 48 h after exposure (**c**), and of the number of necrotic hepatocytes at 168 h (7 days) after exposure (**d**) on applied APAP concentration. Results are based on ten simulations per condition represented as mean ± sd.

Firstly, we questioned how alterations in zonal expression of GSH and P450 affected tissue damage recovery. We compared adversity onset and recovery in lobule simulations with zonation of GSH and P450, in simulations with zonation in one of these molecules, or without zonation (Fig. 3a; Supplementary Movie 2–3). The size of the necrotic area was strongly affected by alterations in the zonation regime. In the base model, in which zonation of P450 and GSH was modeled to resemble experimental data^22,85–88^, the necrotic area gradually increased in size for higher APAP doses (Fig. 3b, purple). Absence of P450 or GSH zonation sensitized the system towards a sharper transition between low and high adversity levels (Fig. 3b, blue, green, yellow), especially upon deletion of the GSH gradient. This steep transition is related to setting the initial intracellular P450 and GSH levels for all cells to the intermediate concentration within hepatocytes that reside approximately halfway along the porto-central axis. Therefore, hepatocytes in the pericentral region had a higher GSH and lower P450 concentration than in a situation with a gradient, and vice versa, hepatocytes in the periportal region had a lower GSH but higher P450 concentration. This led to little NAPQI-cys production at APAP doses <450, and thus less toxicity than with a P450 gradient. However, at APAP doses >450, the low P450 levels in the pericentral area are still sufficiently high to cause necrosis. At such high doses necrosis also takes place in the periportal area because P450 expression is homogeneous across the lobule in the absence of a gradient. Thus, at high APAP doses there is an overall increase in necrosis compared to a lobule with P450 gradient, explaining the sharp transition. A similar argument holds for absence of a GSH gradient.

Zonation was also an important determinant for the extent of senescence and regeneration. The dose at which we observed a high number of senescent cells decreased from 550 with zonation to 400 without any zonation, and the maximum number of senescent cells at 48 h increased in absence of zonation (Fig. 3c). The high number of senescent cells greatly affected the regenerative potential of the lobule, which was indicated by a steepening drop in the number of healthy cells in absence of zonation (Fig. 3d). These simulations show that GSH zonation and to a lesser extent P450 zonation are essential for restricting APAP-induced damage to the liver and for the regenerative capacity after low and intermediate APAP dosing.

### Augmented MDM2-p53 feedback strength reduces senescence and improves regenerative capacity

**Fig. 4:**
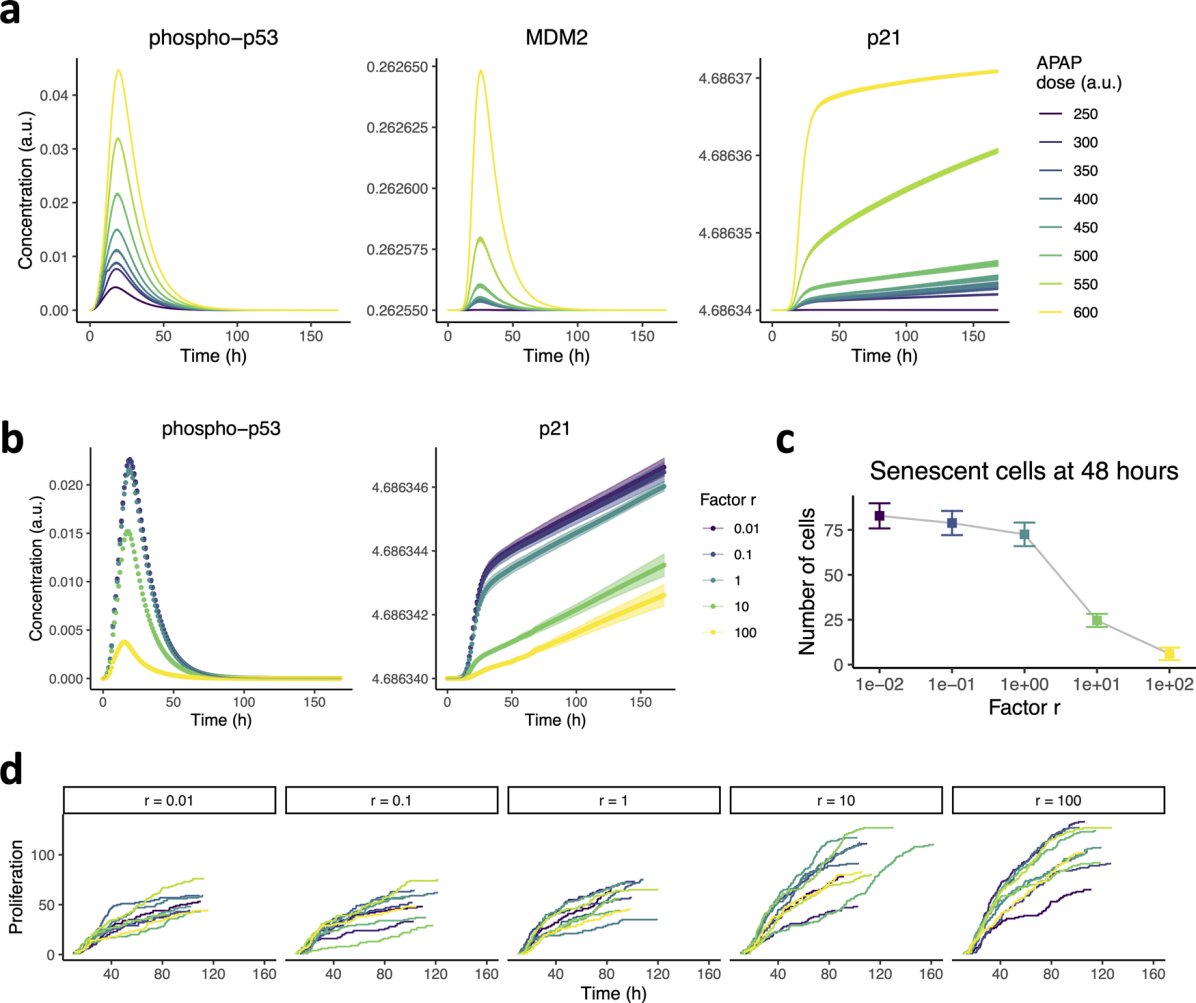
Suppression of DNA damage signaling increases regeneration capacity after APAP insult. **a** Protein expression of phospho-p53, MDM2 and p21 over time after exposure to various APAP concentrations. Due to the deterministic character of the intracellular dynamics, there is very little variation between different simulations per condition. **b** Protein expression of phospho-p53 and p21 over time under different feedback strengths of MDM2 on p53 after exposure to 500 APAP. Factor *r* is the multiplication factor used to scale the MDM2 feedback on p53 and phospho-p53 up or down. Due to the deterministic character of the intracellular dynamics, there is only little variation between different simulations per condition. **c**, **d** The number of senescent cells at 48 h (**c**) and cumulative hepatocyte proliferation events (**d**) after 500 APAP exposure at different MDM2-p53 feedback strengths. Results are based on ten simulations per condition represented as mean ± sd. a.u., arbitrary units.

Next, we investigated whether alterations in the DNA damage response could affect the hepatocellular response to APAP exposure. DNA damage triggers phosphorylation of transcription factor p53 which induces the expression of MDM2 and p21 (Fig. 1b). MDM2 negatively regulates p53 and its phosphorylated form phospho-p53 by targeting it for ubiquitination. In our model simulations, the response of phospho-p53, MDM2 and p21 all increased with APAP concentration (Fig. 4a), as well as the total amount of p53, even though unphosphorylated p53 levels temporarily dropped due to faster p53 phosphorylation compared to its production (Supplementary Fig. 3a).

Expression of p21 continued even after phospho-p53 dropped back to its basal expression level, due to TGF-β that stimulates sustained p21 production. Because p53 induces the expression of p21, p53 influences the likelihood of senescence and proliferation events during regeneration. Previously, we suggested that the feedback strength of MDM2 on p53 could vary among cell types and play a role in interindividual variability^68^. Therefore, we decreased and increased the MDM2 feedback on p53 and phospho-p53 with a factor 10 and 100 and examined the effect of an APAP insult on p21, senescence and recovery from damage. Decreased feedback strength, i.e., a decreased factor *r*, had little effect on p53 stimulation, whereas increased feedback strength caused a marked downregulation of p53, phospho-p53 and therefore also of p21 (Fig. 4b; Supplementary Fig. 3b). The decreased p21 expression due to strong MDM2 feedback on p53 was also reflected by the drop in number of senescent cells at 48 h after exposure (Fig. 4c; Supplementary Movie 4), and by increased proliferation (Fig. 4d). Thus, variability in DNA damage signaling across individuals may have substantial effect on the induced senescence and regeneration potential.

### Stimulation of p21 by macrophage-derived TGF-β affects senescence

**Fig. 5:**
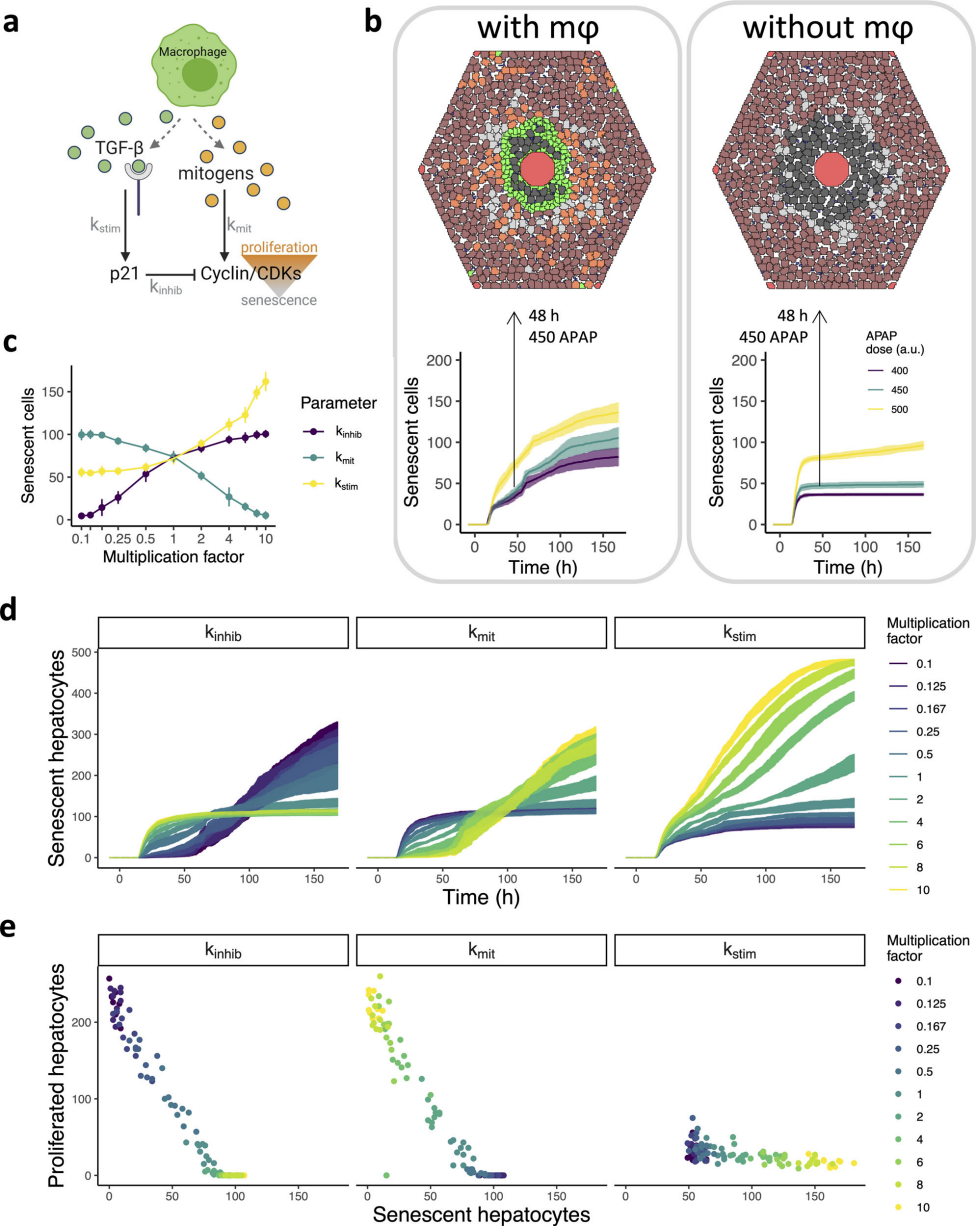
The effect of macrophage-related parameters on senescence and proliferation. **a** Illustration of the reactions and relevant parameters of our model that regulate senescence or proliferation through the cyclin/CDK complex concentration. Macrophages produce TGF-β that stimulate p21 with strength *k*_stim_. The strength of cyclin/CDK inhibition by p21 is regulated by *k*_inhib_. Macrophage-derived mitogens stimulate cyclin/CDKs with strength *k*_mit_. Figure created with BioRender. **b** Bottom panels: The number of senescent cells over time in simulations with and without macrophages after exposure to three APAP concentrations. Top panels: Two stills taken from representative simulations are shown for time point 48 h. Brown, healthy hepatocytes; blue, KCs; red, CV and PVs; black, necrotic hepatocytes; gray, senescent hepatocytes; green, macrophages. **c** The effect of changes in parameter values on the number of senescent cells at time point 48 h after 500 APAP exposure. Multiplication factors were used to scale the strength of the indicated regulatory reactions up or down. **d** Temporal dynamics of the number of senescent hepatocytes following changes in parameter values through multiplication with different scalars after 500 APAP exposure. **e** Relation between the amount of proliferation and senescence events following changes in parameter values through multiplication with different scalars after 500 APAP exposure. Results are based on 10 simulations per condition represented as mean ± sd.

Because macrophages enhance senescence through TGF-β production and proliferation through both mitogen expression and p21-mediated inhibition (Fig. 5a), they need to maintain an intricate balance between these processes to avoid destabilization of undamaged liver tissue. To examine the consequences of cell fate modulation by macrophages, we first ran simulations with and without macrophages. In contrast to the continued growth of senescence until ~4 days after APAP administration in presence of macrophages, the rapid growth in the number of senescent cells already halted after 24 h in the absence of macrophages (Fig. 5b). In our model, the presence of macrophages could double the number of senescent cells.

We further investigated the effect of the three reactions that regulate the abundance of p21 and cyclin/CDK complexes. To this end, we altered the parameters involved in these reactions, i.e., *k*_inhib_, *k*_mit_ and *k*_stim_ (Fig. 5a), by increasing or decreasing them up to a factor 10. Parameters *k*_inhib_ and *k*_mit_ had almost symmetric, inverse effects on the number of senescent cells, i.e., a 10-fold decrease in *k*_inhib_ similarly affected the number of senescence events as a 10-fold increase in *k*_mit_ (Fig. 5c; Supplementary Movie 5–7). We obtained a very different effect on senescence by modulation of *k*_stim_. A 4-fold increase in the TGF-β-stimulated p21 production already caused a stronger increase in senescence than the 1.4-fold increase of senescent cells after a 10-fold increase in *k*_inhib_. In contrast, a decrease in *k*_stim_ did not have a profound effect on the number of senescent cells. The difference between perturbations of *k*_inhib_ and *k*_mit_ on the one hand and *k*_stim_ on the other hand was also apparent from the temporal data on the number of senescent cells (Fig. 5d), and the relation between the number of proliferated and senescent cells (Fig. 5e). Perturbations in *k*_inhib_ and *k*_mit_ caused a delayed senescence response, whereas *k*_stim_ did not qualitatively alter the dynamics (Fig. 5d). In addition, an increase in senescence due to a higher *k*_inhib_ or lower *k*_mit_ value comes at the expense of proliferation, whereas there is no such relation upon alterations in *k*_stim_ (Fig. 5e). In conclusion, these model simulations show that alterations in p21 production have a very different effect on cell fate than variations in the inhibitory strength of p21 on cyclin/CDK complexes.

### Early treatment with NAC or 4MP partially prevents acute liver injury

**Fig. 6:**
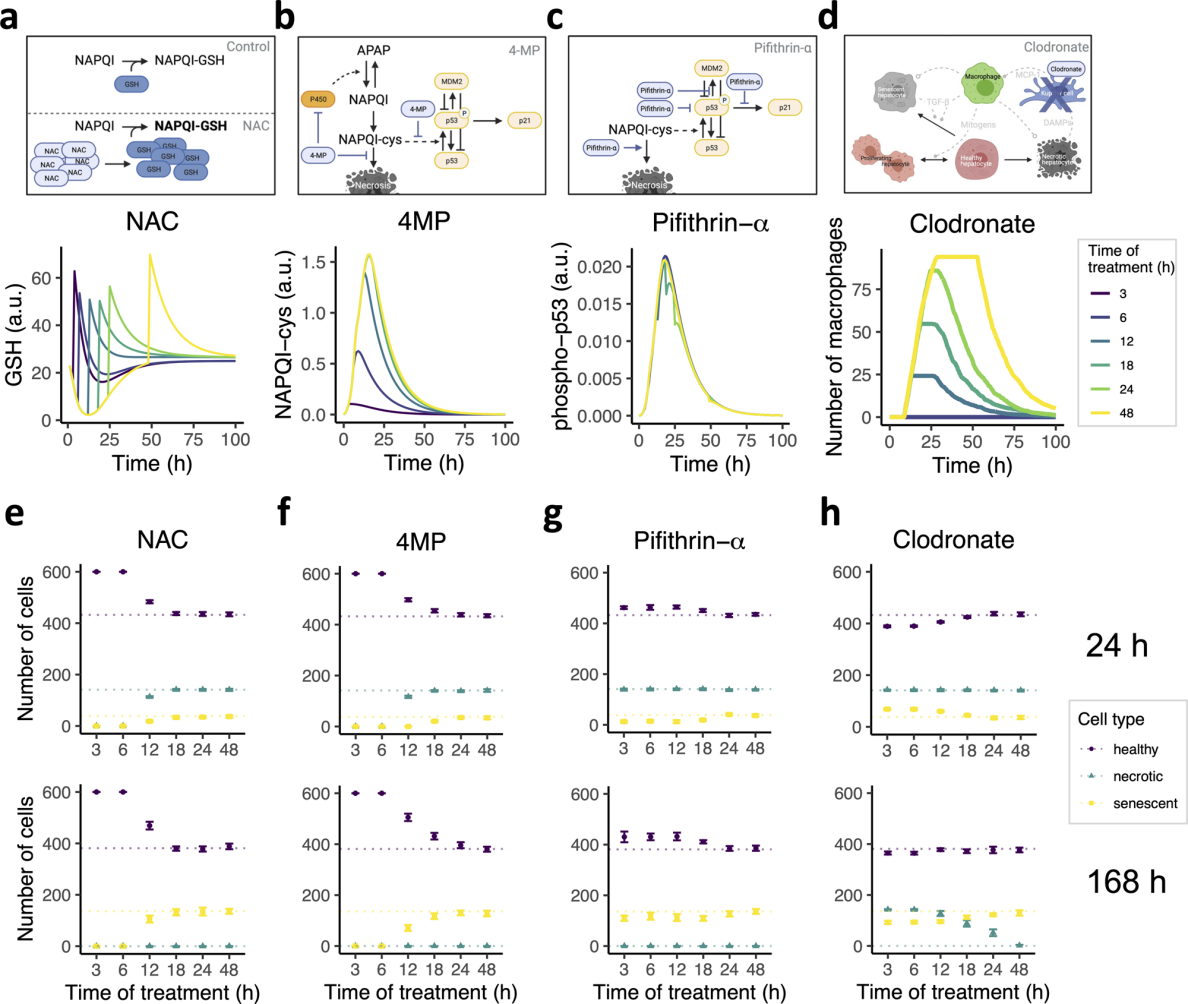
In silico predictions for the effect of four therapies administered at 3, 6, 12, 18, 24, or 48 h on APAP-induced liver injury and recovery. **a**–**d** Graphical representation (top panels) and simulations (bottom panels) of the intracellular effect of (**a**) NAC on GSH concentration, (**b**) 4MP on NAPQI-cys abundance, (**c**) pifithrin-α on phospho-p53 concentration and (**d**) of the effect of KC depletion by clodronate on the number of macrophages. Top panels created with BioRender. **e**–**h** Effect of (**e**) NAC, (**f**) 4MP, (**g**) pifithrin-α and (**h**) clodronate therapies on the healthy, necrotic and senescent cell populations measured at 24 h (top panels) or 168 h (bottom panels). Dotted colored lines indicate simulation results without treatment. Results are based on ten simulations per condition represented as mean ± sd. a.u., arbitrary units.

The model perturbations described above highlight several sources for interindividual variability that likely contribute to susceptibility to adversity. Because of their role in APAP adversity, these processes can also be targeted therapeutically to prevent adversity, and indeed several compounds are frequently used following adverse reaction to APAP^89^. To examine the effect of therapies during different stages of injury initiation and progression on the outcome after injury, we simulated administration of four existing compounds at different time points after APAP exposure at a toxic dose. Firstly, we mimicked the effect of N-acetylcysteine (NAC), a precursor for GSH that detoxifies NAPQI^90,91^, by raising GSH levels with twice the maximal amount of GSH during homeostasis (Fig. 6a). Secondly, we simulated the effect of 4-methylpyrazole (4MP), a compound that inhibits cytochrome P450 activity and activates c-Jun N-terminal kinase (JNK)^92^, thereby directly preventing necrosis and activation of p53. Indeed, the inhibition of P450 activity caused a significant reduction in the amount of NAPQI-cys when given early after APAP exposure (Fig. 6b). Thirdly, we imitated the administration of p53 inhibitor alpha (pifithrin-α) through inhibition of phospho-p53 levels^93^, the transcriptional activity of phospho-p53^94^ and stimulation of necrosis^95^ (Fig. 6c). Lastly, complete depletion of Kupffer cells was used to simulate clodronate administration (Fig. 6d), a compound that specifically depletes Kupffer cells from liver tissue^96,97^.

For each of these compounds, we examined the effect on damage and recovery at 24 (Fig. 6e, f, top panels) and 168 h (Fig. 6e, f, bottom panels) after APAP exposure. Early administration of NAC (Fig. 6e) and 4MP (Fig. 6f), i.e., at 3 or 6 h, could entirely prevent the occurrence of necrosis and senescence, whereas therapies given at 12 h had only moderate beneficial effects compared to no therapy. Moreover, the outcome was not improved for NAC treatments later than 18 h after APAP administration, in contrast to 4MP treatment which had beneficial effects for administration times up to 24 h. Pifithrin-α administration up to 24 h decreased the occurrence of senescence and enhanced the number of healthy cells compared to the control situation without treatment (Fig. 6g), although these effects were small. Note that the slightly altered outcomes were due to the perturbation of p53 signaling and subsequent decrease in p21 expression (Supplementary Fig. 4a–d). Treatment with clodronate had a strong disadvantageous effect on recovery, suggesting that the contribution to regeneration of damaged liver tissue by Kupffer cells and macrophages is more important than their damaging effects due to stimulation of senescence. Specifically, Kupffer cell depletion resulted in limited or absence of necrotic clearance and only moderately reduced the final number of senescent cells compared to a situation without clodronate (Fig. 6h). In conclusion, our simulations of APAP adversity treatment suggest that early treatment with NAC or 4MP may partially prevent toxic outcome, with a slightly longer window of opportunity for 4MP than for NAC.

## Discussion

There is considerable interindividual variability in the susceptibility to and severity of liver injury after APAP overdosing and the likelihood of recovery^98–103^. To gain insight into the factors that determine the extent of injury and recovery potential, we built a computational model that integrates existing models for subcellular biomolecular dynamics with intercellular interactions of cells in the liver lobule. Our simulations showed that regeneration is possible even after profound injury if there are sufficient undamaged hepatocytes. However, the crossing of a tipping point associated with appearance of too many senescent cells prevents successful recovery, a phenomenon that has also been observed in simulations of a Cellular Automata model^65^. Thus, our analysis supports the notion that regulation of senescence is an essential determinant for liver failure^14^. Although our model mimics an ‘average’ individual, it can be used to study sources for interindividual variability. Through systematic perturbations to different parts of the model, we showed that metabolic processes and protein dynamics of individual hepatocytes, as well as signaling between cells can influence the extent of damage. Specifically, we demonstrated that variations in the metabolic pathway primarily affect necrotic damage, whereas intra- and extracellular stress signaling are determinants for the extent of senescence. By targeting these distinct processes with in silico simulations of therapies, we showed that besides early treatment to prevent necrosis, late stage inhibition of senescence through p21 inhibition can improve recovery, although this effect was small.

We found that one important determinant of the extent of damage is lobular zonation. Through alternating removal of zonal differences in P450 and GSH levels, our analysis indicates that GSH zonation is expected to have a stronger effect on the size of necrotic damage than P450 zonation, which originates from the steeper expression gradient of GSH than P450 that we implemented following experimental measurements^85,88,104^. In contrast to our finding, previous research attributed centrilobular necrosis primarily to the high P450 levels in that area^22^. Because the exact distribution profiles of GSH and P450 across the liver lobule are not known, it is difficult to determine what the contribution of these molecules for necrotic damage is. Moreover, the dependency of the GSH replenishment rate on location^105^ and APAP exposure^106^ further complicates obtaining a detailed understanding of the P450 and GSH zonation profile. To unravel the relevance of these necrosis-determining factors in full, spatio-temporal GSH and P450 measurements are needed. Our model can then integrate these data, as well as data on other molecules and metabolic processes that are known to be zonally distributed, such as glucuronidation and sulfation^23,107^.

Another important determinant for lobular damage is the intracellular expression of p21. Deletion of p21 provokes proliferation even in injured hepatocytes^108^, whereas high p21 expression can cause and maintain cell cycle arrest^40,109,110^. To investigate the role of regulatory pathways on senescence and proliferation events after APAP-induced liver injury, we adopted a simple mathematical model for p53 and p21 and coupled it directly to cyclin/CDK complexes involved in cell cycle progression. Our model simulations exhibited sustained p21 protein expression mediated by extracellular TGF-β signaling after the return of p53 to basal levels, irrespective of the MDM2 feedback strength on p53. This contrasted with measurements of p21 mRNA and protein levels that peaked at 6 h and gradually declined until 96 h after exposure to APAP^36^ and a decline in p21-positive cells 2 days after APAP exposure^14^. Because these data originate from population level measurements, single-cell data on p21 expression and its predictive capacity for senescence of hepatocytes at different positions in the lobule would be necessary to determine potential heterogeneity in p21 expression and its relation to dynamics of the entire population. In addition, single-cell quantification of p21 expression after inhibition of either p53 or TGF-β could elucidate the relative importance of the different p21 production stimulants, after which subsequent adjustments in our model might resolve the discrepancy between our simulations and population data.

In addition to the integration of spatial orientation with intracellular molecular dynamics, we incorporated immunosurveillance to adequately model APAP-induced injury and recovery. We modeled KC-dependent recruitment of macrophages, because the latter cells are essential for clearance of senescent and necrotic hepatocytes, and maintenance of the balance between transmission of senescence and promotion of proliferation. However, the interplay between hepatocytes and immune cells is much more complex: besides KCs, hepatocytes with elevated p21 expression also secrete the chemokines CXCL14 and MCP-1, thereby stimulating further recruitment and prolonging the residence of macrophages^111,112^ that clear senescent cells^113,114^. This decreases the senescence-dependent TGF-β production, which limits pro-senescent signaling and therefore could enhance the regenerative capacity of the liver. Thus, immunosurveillance stimulates transmission of senescence^14^, but simultaneously counteracts this transmission by clearance of senescence, which illustrates the intricate balance between the harmful and protective function of senescence in liver damage^115,116^. Furthermore, the presented model does not include regulation of regeneration by Kupffer cells, although KCs do play an essential role in recovery through their production of IL-6, IL-10 and TNF-α^117,118^. In addition, to improve the accuracy of the model for immunosurveillance, representative values for parameters such as the number and maturation time of macrophages, depletion and recovery of the KC population^45,47,53^ and immune cell-specific production and diffusion of chemokines are necessary. Because we derived values for some of the parameters through comparison of simulations to published system outcomes in mice, we propose to experimentally obtain accurate estimates for currently unknown parameter values while in parallel extending the model with the discussed additional processes involved in liver recovery.

We used our model to predict the effect of four therapeutical compounds administered at different time points after APAP exposure. In our simulations, administration of NAC or 4MP within 6 h prevented necrotic damage. Indeed, NAC treatment has a protective effect at least until 8 h after APAP exposure^119–121^ and 4MP has been suggested to have a larger therapeutic window in APAP-exposed human hepatocytes than NAC^89,122^, which corresponds to our results. With respect to the effects of pifithrin-α and clodronate administration, our in silico simulations predicted mild reductions in senescence. However, the co-occurring rise in necrosis as described in literature^95,97,117^ did not occur in our model, possibly because stimulation of necrosis was exclusively determined by NAPQI-cys abundance. Extension of the model with intermediary JNK that amplifies necrosis together with negative feedback from phospho-p53 to JNK and faster necrotic clearance by macrophages, complemented by experimental time-resolved data to ensure adequate parameterization, would further improve predictions for necrosis. Because of the interference of p53 and macrophages with necrosis, direct inhibition of p21 or indirect inhibition via obstruction of TGF-β signaling, could be a more promising approach to prevent senescence and circumvent increased necrosis. In addition, promotion rather than depletion of macrophages could have beneficial effects for ALI outcome^50^. Experimental data on the extent of necrosis, senescence and regeneration, and intracellular protein signaling measurements after administration of the modeled therapies at different doses and time points after APAP exposure, would be useful to improve the parameterization of these therapies. Moreover, such measurements would allow to verify the effect of these therapies on the outcome or detect crucial components that are currently not included in the model. Although our model currently lacks some processes that may be important to adequately predict the effect of therapeutic interventions, our results clearly indicate that prevention of NAPQI-cys formation is most effective to limit hepatocellular damage. Furthermore, we hypothesize that late-stage inhibition of p21 and stimulation of macrophage recruitment have beneficial effects at late stages beyond APAP exposure when most necrosis has already occurred.

Examining the effect of perturbations of various processes in our model revealed critical components that are potential sources of interindividual variability in outcome. An important next step is to quantify the relation between the extent of necrosis, senescence and regeneration with the probability of organismal survival or death. Mice do not all die or all stay alive after exposure to equal amounts of APAP that results in a similar extent of necrosis^25^, demonstrating the complexity of this relation. Existing models to predict mortality make use of damage biomarkers in plasma^61,123^, but other factors such as the extent of senescence^14,124^, potential of regeneration^74^ and effectivity of the immune response^125^ also contribute to the interindividual difference in susceptibility to liver failure and death. Therefore, accurate mortality predictions are not straightforward and misclassification by models is common. Nevertheless, a threshold of 70% hepatic necrosis has been previously used to differentiate recovery from death with satisfying results^61^. Another study reported a maximum of 40% necrosis at 600 mg/kg in mice which was lethal for 25% of the animals^35^, illustrating the interindividual variability in susceptibility to a lethal outcome. Whereas the extreme outcomes of our simulations with only damaged hepatocytes left, i.e., at doses of 600 APAP and higher, could be considered as organism death, it is not straightforward how our model outcomes at moderately high doses relate to the probability of lethality. Additional research should elucidate how the extent of necrosis, senescence, potential for regeneration, the immune response and other important factors affect the probability of survival.

Given the current extensive knowledge on the adverse outcome pathway and the existence of numerous models for APAP-induced liver injury, we aimed to incorporate the essential processes that span different levels of biological organization into one comprehensive model and investigate the sensitivity of the system to changes in several key processes and test its predictive potential. To this end, we used measurements and mechanistic information for metabolism, intra- and extracellular signaling, hepatocyte damage and recovery from multiple existing models and data sources. In contrast to other models that focus on hepatocyte death and survival^61,62,64,65^ without consideration of senescence and immunosurveillance, the interplay between senescence and regeneration, partly regulated by the immune system, is a central part of our model. We performed model perturbations to simulate the effect of interindividual variability and therapies. Overall, the presented model was in good accordance with available data, and we showed that regulation of subcellular as well as intercellular signaling are important determinants for the system’s outcome. However, several improvements based on future experimental data could be made to further elucidate and validate the mechanisms behind onset of senescence and proliferative events, and the probability of survival. In addition, alterations in the lobular structure of the model or replacement of specific modules, such as the module for drug metabolism, would make it applicable to other organisms or drugs. Thus, our approach provides an excellent basis to be exploited for future in silico drug adversity research.

## Methods

### Cellular Potts model

To model spatiotemporal dynamics of liver lobule cellular interactions and intracellular acetaminophen metabolism and protein dynamics, we used a two-dimensional cellular Potts model (CPM)^69,70^ in combination with differential equation modeling. In the CPM formalism, cells are represented as adjacent sites on a lattice with the same cell ID *σ* and that can extend at each step by occupying a neighboring lattice site with a different cell ID *σ*′. Such an extension occurs with a Monte Carlo probability of 1 if the change in the Hamiltonian energy function (Δ*H*) is <0, and a Monte Carlo probability dependent on Δ*H* if Δ*H* is >0, i.e.,

1 $${{{\mathrm{P}}}}\left( {\sigma \to \sigma^\prime } \right) = \left\{ {\begin{array}{*{20}{c}} {1;\,{\Delta}{{{\mathrm{H}}}}\, < \,0} \\ {{{{\mathrm{e}}}}^{ - \frac{{{\Delta}{{{\mathrm{H}}}}}}{{{{\mathrm{T}}}}}};\,{\Delta}{{{\mathrm{H}}}} \ge 0}, \end{array}} \right.$$

with simulation temperature *T*^69^. The basic Hamiltonian consists of energy terms that describe cell adhesion and an area constraint, but this can be extended with processes such as chemotaxis:

2 $${{{\mathrm{H}}}} = \mathop {\sum}\limits_{ij} {\mathop {\sum}\limits_{i^\prime j^\prime } {{{{\mathrm{J}}}}_{\tau (\sigma _{ij}),\tau (\sigma _{i^\prime j^\prime })}} } \left( {1 - \delta _{\sigma _{ij},\sigma _{i^\prime j^\prime }}} \right) + \mathop {\sum}\limits_\sigma {\lambda _a} ({{{\mathrm{a}}}}_\sigma - {{{\mathrm{A}}}}_\sigma )^2 - \mu _\sigma \cdot {{{\mathrm{c}}}},$$

where J represents the surface energy between cells *σ*_*i,j*_ and *σ*_*i*′*,j*′_ at location coordinates *i*,*j* and of cell type *τ*, *δ* the Kronecker delta, *λ* the weight of the difference between the actual area *a*_*σ*_ and target area *A*_*σ*_, *μ*_*σ*_ the chemotactic strength and *c* the concentration of the chemoattractant^126,127^. Note that we applied the chemotaxis term only to the in silico macrophages, and for the other modeled cell types we only applied the first two terms. We complemented the spatial model with partial and ordinary differential equations, to model extracellular species’ concentrations and intracellular molecular dynamics. We used published models and accompanying parameter values as base for our model, making adjustments where needed (see below). Model parameter values that have not been described previously were chosen such that simulation read-outs matched findings reported by reference studies. The two-dimensional CPM of a liver lobule together with the molecular dynamics model was implemented in the modeling and simulation environment Morpheus, version 2.2.0^128^.

### Liver lobule composition and model

We initiated a square grid of 200 × 200 pixels in which we defined a hexagonal region with six edges as the model domain. The hexagonal vertices were placed at the coordinates provided in Supplementary Table 2. Because of the limited amount of quantified human liver characteristics, we used data of the lobule composition of rodents (mice and rats) as basis for the spatial model, although a liver lobule in humans is slightly larger than in rodents^129^. The lobular radius in mice has been reported to be on average 284 μm^64^, accounting for a node length of 3 μm/pixel in our model domain with a radius of 95 pixels. Because the diameter of portal veins at the lobule corners ranges from 15–35 μm^130^, we placed six circular entities with a 5-pixel radius centered at the vertex coordinates. A circular central vein CV with a 41 μm radius^64^ equivalent to 14 pixels in our model, was centered at the coordinates (100,100), i.e., the center of the hexagon. Excluding the PV and CV radii, the distance between the PV and CV is about 228 μm, and with a hepatocyte diameter of 17–23 μm^64,131^ the porto-central axis contains 10 to 14 hepatocytes. In addition, about 80% of the liver volume and 60% of total liver cells consists of hepatocytes and only about 2% of the volume and 15% of the cells are Kupffer cells^132,133^, i.e., a ten-fold difference in volume per cell. Note that these percentages are derived from rat liver tissue. Based on these ratios, we initiated 600 hepatocytes and 150 Kupffer cells to account for the four-fold difference in cell numbers. In addition, we set the target area at 35 pixels for hepatocytes of 20 μm in diameter (considering circular cells) and set a ten times smaller target area for Kupffer cells, i.e., 3.5 pixels. Note that the low target area for Kupffer cells did not lead to their frequent disappearance during simulations due to attainment of an area of zero pixels by chance. Hepatocytes and Kupffer cells were initiated as single pixels at random positions within a circular region with a radius of 80 pixels around the lattice center. Macrophages were considered to be half the size of a healthy hepatocyte^134,135^, i.e., with a target area of 17.5 pixels, and were initially absent from the simulation. Instead of explicitly modeling the sinusoidal structures, intercellular spaces and additional cell types such as endothelial and fat-storing cells^133^, empty lattice sites represent such entities. Note that the absence of a fixed network of sinusoids in our model likely caused a higher migration potential for in silico hepatocytes than in reality or in a model with a fixed sinusoidal structure. Because increased migration causes the neighbors of hepatocytes to vary over time and thereby reduces communication time between cells, we chose interaction parameters such that we still observe realistic proliferation and transmission of senescence. An overview of surface energies, target cell areas and area constraints can be found in Supplementary Table 3.

### APAP distribution and hepatocellular metabolism

To accurately model single-cell exposure to APAP and its toxic metabolites, we modeled APAP exposure, distribution, uptake, metabolism and excretion as distinct processes. APAP exposure started after 96 Monte Carlo steps (MCSs), to ensure completion of an initialization phase to a steady state of hepatocyte and Kupffer cell size and spatial distribution. APAP concentration within the in silico liver lobule was modeled as a scalar field. Because blood flow velocity within a lobule ranges from a few to about 300 μm/s^63,136,137^, we assumed that APAP fully spreads within a few seconds throughout the sinusoidal network. Compared to the temporal scale of 5 min per MCS as used in our model, this distribution time is negligible. Therefore, we considered a homogeneous distribution of APAP across the liver lobule. The majority of APAP in the blood is taken up by the liver and only about 2–5% is directly excreted via the urine^16,19,138^. The change in APAP plasma concentration (*P*_*ex*_) over time (in hours) was therefore modeled by:

3 $$\frac{{{{{\mathrm{dP}}}}_{{{{\mathrm{ex}}}}}}}{{{{{\mathrm{dt}}}}}} = - {{{\mathrm{k}}}}_{{{\mathrm{u}}}} \cdot {{{\mathrm{P}}}}_{{{{\mathrm{local}}}}} - {{{\mathrm{k}}}}_{{{\mathrm{e}}}} \cdot {{{\mathrm{P}}}}_{{{{\mathrm{ex}}}}},$$

where *P*_*local*_ is the mean APAP blood concentration at the site of a hepatocyte, uptake (*k*_*u*_) and excretion (*k*_*e*_) parameters were chosen such that the APAP half-life in the model corresponded to values reported in literature, i.e., about 2–3 h^18,77–80^. We used an existing APAP metabolism model that incorporates sulfation by PAPS, glucuronidation, oxidation to NAPQI by cytochrome P450 and detoxification by GSH^58^ to model APAP metabolism within every single hepatocyte separately. We added cytochrome P450 (*P*450) as static species to this model to account for its zonal distribution. In addition, we added a degradation term for NAPQI-cys adducts to account for adduct elimination^106,139^ and allow cellular recovery. This resulted in the following equations for intracellular APAP (*P*_*in*_), PAPS (*S*), NAPQI (*N*), GSH (*G*) and NAPQI-cys adducts (*C*):

4 $$\frac{{{{{\mathrm{dP}}}}_{{{{\mathrm{in}}}}}}}{{{{{\mathrm{dt}}}}}} = {{{\mathrm{k}}}}_{{{\mathrm{u}}}} \cdot {{{\mathrm{P}}}}_{{{{\mathrm{local}}}}} - {{{\mathrm{k}}}}_{{{\mathrm{s}}}} \cdot {{{\mathrm{S}}}} \cdot {{{\mathrm{P}}}}_{{{{\mathrm{in}}}}} - {{{\mathrm{k}}}}_{{{\mathrm{g}}}} \cdot {{{\mathrm{P}}}}_{{{{\mathrm{in}}}}} - {{{\mathrm{k}}}}_{{{{\mathrm{P}}}}450} \cdot {{{\mathrm{P}}}}_{{{{\mathrm{in}}}}} \cdot {{{\mathrm{P}}}}450 + {{{\mathrm{k}}}}_{{{\mathrm{N}}}} \cdot {{{\mathrm{N}}}},$$

5 $$\frac{{{{{\mathrm{dS}}}}}}{{{{{\mathrm{dt}}}}}} = - {{{\mathrm{k}}}}_{{{\mathrm{s}}}} \cdot {{{\mathrm{S}}}} \cdot {{{\mathrm{P}}}}_{{{{\mathrm{in}}}}} + {{{\mathrm{b}}}}_{{{\mathrm{S}}}} - {{{\mathrm{d}}}}_{{{\mathrm{S}}}} \cdot {{{\mathrm{S,}}}}$$

6 $$\frac{{{{{\mathrm{dN}}}}}}{{{{{\mathrm{dt}}}}}} = {{{\mathrm{k}}}}_{{{{\mathrm{P}}}}450} \cdot {{{\mathrm{P}}}}_{{{{\mathrm{in}}}}} \cdot {{{\mathrm{P}}}}450 - {{{\mathrm{k}}}}_{{{\mathrm{N}}}} \cdot {{{\mathrm{N}}}} - {{{\mathrm{k}}}}_{{{{\mathrm{GSH}}}}} \cdot {{{\mathrm{N}}}} \cdot {{{\mathrm{G}}}} - {{{\mathrm{k}}}}_{{{{\mathrm{PSH}}}}} \cdot {{{\mathrm{N,}}}}$$

7 $$\frac{{{{{\mathrm{dG}}}}}}{{{{{\mathrm{dt}}}}}} = - {{{\mathrm{k}}}}_{{{{\mathrm{GSH}}}}} \cdot {{{\mathrm{N}}}} \cdot {{{\mathrm{G}}}} + {{{\mathrm{b}}}}_{{{{\mathrm{GSH}}}}} - {{{\mathrm{d}}}}_{{{{\mathrm{GSH}}}}} \cdot {{{\mathrm{G,}}}}$$

8 $$\frac{{{{{\mathrm{dC}}}}}}{{{{{\mathrm{dt}}}}}} = {{{\mathrm{k}}}}_{{{{\mathrm{PSH}}}}} \cdot {{{\mathrm{N}}}} - {{{\mathrm{d}}}}_{{{\mathrm{C}}}} \cdot {{{\mathrm{C}}}}.$$

Parameterization of the model of APAP metabolism was based on previously established values^58^ and adapted to match our time unit, i.e. hours, and account for variable initial concentrations due to zonation. Biological interpretation of parameters and their values are provided in Supplementary Table 4.

### Necrotic cell death and macrophage recruitment

Accumulation of intracellular NAPQI-protein adducts can cause necrotic cell death. In our model, hepatocytes with a too high adduct concentration (*C* > 4) became necrotic cells with probability *p*_necrosis_ = 0.8. Necrotic hepatocytes no longer metabolized APAP and could no longer migrate across the lattice. DAMPs (*D*) released by necrotic cells with rate *r*_D_ = 6 a.u. h^−1^, diffused across the lattice with diffusion constant ${{{\mathcal{D}}}}_{{{\mathrm{D}}}} = 216\,{{{\mathrm{\mu m}}}}^2\,{{{\mathrm{h}}}}^{ - 1}$ and degraded with rate *d*_D_ = 1.2 h^−1^, which we modeled with the partial differential equation:

9 $$\frac{{\partial {{{\mathrm{D}}}}}}{{\partial {{{\mathrm{t}}}}}} = {{{\mathcal{D}}}}_{{{\mathrm{D}}}}\nabla ^2{{{\mathrm{D}}}} + {{{\mathrm{r}}}}_{{{\mathrm{D}}}} - {{{\mathrm{d}}}}_{{{\mathrm{D}}}} \cdot {{{\mathrm{D}}}}.$$

Kupffer cells that sensed a local DAMP concentration >0.5, started to release MCP-1 (*M*) with rate *r*_M_ = 900 h^−1^. We set the diffusion constant of MCP-1 at ${{{\mathcal{D}}}}_{{{\mathrm{M}}}} = 27 \cdot 10^3{{{\mathrm{\mu m}}}}^2\,{{{\mathrm{h}}}}^{ - 1}$ and its degradation rate at *d*_M_ = 2.4 h^−1^, such that MCP-1 rapidly reached the boundaries of the lattice:

10 $$\frac{{\partial {{{\mathrm{M}}}}}}{{\partial {{{\mathrm{t}}}}}} = {{{\mathcal{D}}}}_{{{\mathrm{M}}}}\nabla ^2{{{\mathrm{M}}}} + {{{\mathrm{r}}}}_{{{\mathrm{M}}}} - {{{\mathrm{d}}}}_{{{\mathrm{M}}}} \cdot {{{\mathrm{M}}}}.$$

If the mean MCP-1 concentration in the periportal area exceeded 0.0001 a.u., macrophages appeared as single pixels with probability *p*_mφ_ = 0.5 within the periportal area and quickly grew to their target areas during subsequent simulation time steps. Here, the periportal area was defined as the region spanned by the pixels directly adjacent to the portal vein. To prevent an excess of macrophages from entering the lobule, macrophage recruitment stopped when the mean MCP-1 concentration in the periportal area had substantially decreased at late time points, i.e., when *M*_t = i_ < 0.9 · *M*_max_, where *i* is a time point >0 and *M*_max_ is the maximum MCP-1 concentration in the periportal area achieved during the preceding time period. After entry into the liver lobule, macrophages were attracted chemotactically to regions of high MCP-1 concentration, i.e., to regions with many necrotic cells. The chemotactic strength acting on macrophages within the periportal area was set to *μ*_mφ → M_ = 600, within the neighborhood of necrotic cells to *μ*_mφ → M_ = 2 and elsewhere to *μ*_mφ → M_ = 100. Macrophages released mitogens at rate *r*_mit_ = 48 a.u. h^−1^, that diffused across the lattice with diffusion constant ${{{\mathcal{D}}}}_{{{{\mathrm{MIT}}}}} = 27 \cdot 10^3{{{\mathrm{\mu m}}}}^2{{{\mathrm{h}}}}^{ - 1}$and that were degraded with rate *d*_mit_ = 3.24 h^−1^, following the equation:

11 $$\frac{{\partial {{{\mathrm{MIT}}}}}}{{\partial {{{\mathrm{t}}}}}} = {{{\mathcal{D}}}}_{{{{\mathrm{MIT}}}}}\nabla ^2{{{\mathrm{MIT}}}} + {{{\mathrm{r}}}}_{{{{\mathrm{mit}}}}} - {{{\mathrm{d}}}}_{{{{\mathrm{mit}}}}} \cdot {{{\mathrm{MIT}}}}.$$

Near necrotic cells, i.e., within the Moore neighborhood of 8 pixels, macrophages also released TGF-β (*T*) at rate *t*_mφ_ = 2.4 a.u. h^−1^. A necrotic cell was cleared by phagocytosis with a probability *p*_phagocytosis_ = 0.1 when it had been in contact with a macrophage for over 750 min and was in the neighborhood of a macrophage. Because MCP-1 was produced by Kupffer cells in the vicinity of necrotic hepatocytes, MCP-1 levels dropped rapidly after necrotic clearance. Macrophages disappeared with a probability *p*_mφ retreat_ = 0.005 when their elapsed lifespan was >1000 min and the MCP-1 concentration in the periportal area was <1·10^−5^ a.u. Note that previous studies^14,47^ showed a gradual decrease of macrophages until 5 days after administration, whereas in our model the number of macrophages did not decrease until clearance of necrotic debris was completed because we did not include a process to actively remove macrophages.

### DNA damage response activation

To model the impact of NAPQI-cys and the resulting oxidative stress induced nuclear DNA damage^140,141^ on subsequent cellular demise, we adapted our previously created DNA damage model^68^ and incorporated the ODE equations into our intracellular dynamics model of the hepatocytes. Specifically, we removed the mRNA state variables from the model and re-estimated the parameters of this reduced model (Supplementary Fig. 1 and Supplementary Methods). For re-estimation of the parameters, we employed our previously established in-house derivative-based optimization method, that uses the least squares minimization function from the SciPy package in Python version 3.7.3 in combination with Latin hypercube sampling for efficient sampling of the parameter space^68^. Subsequently, we further adapted this reduced model to integrate it in our spatial model, as described below. The incorporated equations included those for p53 (*P53*) and its phosphorylated state (*P53*_*p*_), the downstream target as well as negative regulator MDM2 (*MDM2*), and target p21 (*P21*) which plays an important role in cell cycle arrest. We made the p53 phosphorylation rate directly dependent on the amount of NAPQI-cys within a cell. In addition, we added a stimulatory term to the equation of p21 that depended on the proportion of occupied TGF-β receptors (*RT*) and rate constant *k*_stim_ = 12·10^−6^ h^−1^. This resulted in Eqs. 12–15, in which the time-dependent parameter values were modified to match the time units of our spatial model (see Supplementary Table 5):

12 $$\frac{{{{{\mathrm{dP}}}}53}}{{{{{\mathrm{dt}}}}}} = {{{\mathrm{ks}}}}_{{{{\mathrm{p}}}}53} + {{{\mathrm{k}}}}_{{{{\mathrm{dp}}}}} \cdot {{{\mathrm{P}}}}53_{{{\mathrm{p}}}} - {{{\mathrm{k}}}}_{{{\mathrm{p}}}} \cdot {{{\mathrm{P}}}}53 \cdot {{{\mathrm{C}}}} - {{{\mathrm{kd}}}}_{{{{\mathrm{p}}}}53} \cdot {{{\mathrm{P}}}}53 - {{{\mathrm{kd}}}}_{{{{\mathrm{p}}}}53\,{{{\mathrm{mdm}}}}2} \cdot {{{\mathrm{P}}}}53 \cdot {{{\mathrm{MDM}}}}2,$$

13 $$\frac{{{{{\mathrm{dP}}}}53_{{{\mathrm{p}}}}}}{{{{{\mathrm{dt}}}}}} = {{{\mathrm{k}}}}_{{{\mathrm{p}}}} \cdot {{{\mathrm{P}}}}53 \cdot {{{\mathrm{C}}}} - {{{\mathrm{k}}}}_{{{{\mathrm{dp}}}}} \cdot {{{\mathrm{P}}}}53_{{{\mathrm{p}}}} - {{{\mathrm{kd}}}}_{{{{\mathrm{p}}}}53{{{\mathrm{p}}}}} \cdot {{{\mathrm{P}}}}53_{{{\mathrm{p}}}} - {{{\mathrm{kd}}}}_{{{{\mathrm{p}}}}53{{{\mathrm{p}}}}\,{{{\mathrm{mdm}}}}2} \cdot {{{\mathrm{P}}}}53_{{{\mathrm{p}}}} \cdot {{{\mathrm{MDM}}}}2,$$

14 $$\frac{{{{{\mathrm{dMDM}}}}2}}{{{{{\mathrm{dt}}}}}} = {{{\mathrm{ks}}}}_{{{{\mathrm{mdm}}}}2} + \frac{{{{{\mathrm{ks}}}}_{{{{\mathrm{mdm}}}}2\;{{{\mathrm{p}}}}53{{{\mathrm{p}}}}} \cdot {{{\mathrm{P}}}}53_{{{\mathrm{p}}}}^4}}{{{{{\mathrm{Km}}}}_{{{{\mathrm{mdm}}}}2}^4 + {{{\mathrm{P}}}}53_{{{\mathrm{p}}}}^4}} - {{{\mathrm{kd}}}}_{{{{\mathrm{mdm}}}}2} \cdot {{{\mathrm{MDM}}}}2$$

and

15 $$\frac{{{{{\mathrm{dP}}}}21}}{{{{{\mathrm{dt}}}}}} = {{{\mathrm{ks}}}}_{{{{\mathrm{p}}}}21} + \frac{{{{{\mathrm{ks}}}}_{{{{\mathrm{p}}}}21\;{{{\mathrm{p}}}}53{{{\mathrm{p}}}}} \cdot {{{\mathrm{P}}}}53_{{{\mathrm{p}}}}^4}}{{{{{\mathrm{Km}}}}_{{{{\mathrm{p}}}}21}^4 + {{{\mathrm{P}}}}53_{{{\mathrm{p}}}}^4}} - {{{\mathrm{kd}}}}_{{{{\mathrm{p}}}}21} \cdot {{{\mathrm{P}}}}21 + {{{\mathrm{k}}}}_{{{{\mathrm{stim}}}}} \cdot {{{\mathrm{RT}}}},$$

We modeled the proportion of free (*R*) and occupied (*RT*) TGF-β receptors with

16 $$\frac{{{{{\mathrm{dR}}}}}}{{{{{\mathrm{dt}}}}}} = {{{\mathrm{k}}}}_{{{{\mathrm{off}}}}} \cdot {{{\mathrm{RT}}}} - {{{\mathrm{k}}}}_{{{{\mathrm{on}}}}} \cdot {{{\mathrm{T}}}} \cdot {{{\mathrm{R}}}}$$

and

17 $$\frac{{{{{\mathrm{dRT}}}}}}{{{{{\mathrm{dt}}}}}} = {{{\mathrm{k}}}}_{{{{\mathrm{on}}}}} \cdot {{{\mathrm{T}}}} \cdot {{{\mathrm{R}}}} - {{{\mathrm{k}}}}_{{{{\mathrm{off}}}}} \cdot {{{\mathrm{RT}}}},$$

where *k*_on_ = 3 a.u.^−1^h^−1^ and *k*_off_ = 6 h^−1^ were the association and dissociation rates between TGF-β and its receptor. We described the TGF-β concentration with

18 $$\frac{{\partial {{{\mathrm{T}}}}}}{{\partial {{{\mathrm{t}}}}}} = {{{\mathcal{D}}}}_{{{\mathrm{T}}}}\nabla ^2{{{\mathrm{T}}}} + {{{\mathrm{t}}}}_{{{{\mathrm{macrophage}}}}} + {{{\mathrm{t}}}}_{{{{\mathrm{senescent}}}}} + {{{\mathrm{k}}}}_{{{{\mathrm{on}}}}} \cdot {{{\mathrm{T}}}} \cdot {{{\mathrm{R}}}} - {{{\mathrm{k}}}}_{{{{\mathrm{off}}}}} \cdot {{{\mathrm{RT}}}} - {{{\mathrm{d}}}}_{{{\mathrm{T}}}} \cdot {{{\mathrm{T}}}},$$

in which ${{{\mathcal{D}}}}_{{{\mathrm{T}}}}\nabla ^2{{{\mathrm{T}}}}$ represented the diffusion of extracellular TGF-β with diffusion constant ${{{\mathcal{D}}}}_{{{\mathrm{T}}}}$, *t*_macrophage_ = 2.4 a.u. h^−1^ and *t*_senescent_ = 0.12 a.u. h^−1^ the macrophage- and senescent cell-dependent TGF-β production rates, and *d*_T_ = 3.6 h^−1^ the degradation rate of TGF-β.

### Regulation of senescence and proliferation

In our model, the transition from healthy, non-proliferating hepatocytes into either a proliferating or a senescent hepatocyte depended on the intracellular concentration of cyclin/CDK complexes (*K*). The abundance of these complexes was regulated by basal production rate constant *b*_k_ and degradation constant *d*_k_ = 3.35·10^−3^ h^−1^, and these processes were enhanced by local presence of macrophage-derived mitogenic signals and p21-dependent inhibition, as follows:

19 $$\frac{{{{{\mathrm{dK}}}}}}{{{{{\mathrm{dt}}}}}} = {{{\mathrm{b}}}}_{{{\mathrm{K}}}} - {{{\mathrm{d}}}}_{{{\mathrm{K}}}} \cdot {{{\mathrm{K}}}} + {{{\mathrm{k}}}}_{{{{\mathrm{mit}}}}} \cdot {{{\mathrm{MIT}}}}_{{{{\mathrm{local}}}}} - {{{\mathrm{k}}}}_{{{{\mathrm{inhib}}}}} \cdot {{{\mathrm{P}}}}21 \cdot {{{\mathrm{K}}}},$$

with *k*_mit_ = 6.24·10^−4^ h^−1^, *k*_inhib_ = 24 a.u.^−1^ h^−1^, and b_k_ defined by steady state constraint *b*_k_ = *d*_K_ · *K*_initial state_ + *k*_inhib_ · *P21*_initial state_ · *K*_initial state_. Hepatocytes proliferated with probability *p*_proliferation_ = 0.001 when the intracellular cyclin/CDK concentration *K* > 4.68641 a.u., and went into an irreversible senescent state when *K* < 4.6863375 a.u.. A senescent cell was cleared with a probability of 0.45 if it resided in the Moore neighborhood of a macrophage for >300 min. Note that in the absence of macrophages, senescent cells are not removed and remain in this state.

### Simulation runs and model perturbations

We initiated and ran every model condition ten times, to account for the variability in simulation outcomes due to the partially stochastic nature of some model processes. To investigate the sensitivity of the liver lobule to alterations in GSH and P450 zonation strengths, we removed the dependency of GSH levels, P450 levels or both on the cell distance to the central vein. For the simulations without zonation, we set the initial amount of GSH and P450 for all hepatocytes at an intermediate level, i.e., at the initial level in a cell halfway between the CV and the lobule border, which was 20.61 a.u. for GSH and 1.15 a.u. for P450. For the simulations with different MDM2-p53 feedback strengths, we altered the feedback by multiplying the original parameter value with *r* ∈ {0.01, 0.1} for weakened, and *r* ∈ {10, 100} for augmented feedback strength. Similarly, to investigate the importance of the parameters related to cyclin/CDK complex regulation, we multiplied the original values for *k*_inhib_, *k*_mit_ and *k*_stim_ with parameter multiplication factors from the set {0.1, 0.125, 0.167, 0.25, 0.5, 1, 2, 4, 6, 8, 10}. Complete removal of macrophages was achieved by setting the probability of macrophage recruitment to *p*_mφ_ = 0.

### Simulations of treatment with therapies

We simulated a treatment with four different therapies at 3, 6, 12, 18, 24 and 48 h after APAP administration. For simulation of NAC therapy that increases the amount of GSH, we added twice the maximum GSH concentration attained during the time period preceding APAP exposure (considering all hepatocytes in the lobule) to the GSH concentration at the time of treatment, corresponding to values used in a previous study^90^.

To model the effect of 4MP, we considered the different target sites of 4MP. First, because 4MP causes considerable phospho-JNK inhibition which negatively impacts p53 activation and necrosis^92^, we considered that 4MP causes a conservative 80% decrease of p53 activation and simulated this by multiplying the phosphorylation rate of p53 with parameter *k*_4MP JNK_ = 0.2 (i.e., decreasing the phosphorylation rate). Second, because our model does not contain negative feedback from phospho-p53 to JNK and JNK-dependent stimulation of necrosis, we also included direct inhibition from 4MP on necrosis by decreasing the probability for a necrotic event to a small but non-negligible *p*_necrosis_ = 0.00005. Third, we lowered the amount of P450 with 90% through multiplication of P450 with *k*_4MP P450_ = 0.1, because 4MP was previously reported to cause a dramatic reduction of ~90% in NAPQI-cys adducts^142^.

We simulated the effect of pifithrin-α treatment by multiplying the phospho-p53-dependent production of p21 and MDM2 with *k*_PFT_ = 0.5, and the amount of phospho-p53 and the phosphorylation rate of p53 with *k*_PFT phos_ = 0.67 at the time of treatment, because the effect of pifithrin-α causes approximately a 50% decrease in p53-dependent transcriptional activation^95^ and a 33% decrease in phosphorylated p53 species^93^. In addition, we set *p*_necrosis_ = 1 to account for the amplified necrosis that results from phospho-p53 inhibition (due to JNK activation). We simulated the depletion of Kupffer cells by eliminating all KCs at the time of treatment.

## Supplementary information

Supplementary Information

Supplementary Movie 1

Supplementary Movie 2

Supplementary Movie 3

Supplementary Movie 4

Supplementary Movie 5

Supplementary Movie 6

Supplementary Movie 7

## Data Availability

All model simulation results are available via the persistent link 10.5281/zenodo.6651479.

## Code availability

All models and code for data analysis, model simulations and figure generation are available via the persistent link 10.5281/zenodo.6651479.
