# Supplementary material for: Unraveling the effect of intra- and intercellular processes on acetaminophen-induced liver injury
Source: NPJ Syst Biol Appl. 2022 Aug 6;8:27. doi: 10.1038/s41540-022-00238-5 (PMC9357019; doi:10.1038/s41540-022-00238-5)
Supplement: Supplementary file 1 — Supplementary Information [file 41540_2022_238_MOESM1_ESM.pdf]

Supplementary Information

Unraveling the effect of intra- and intercellular processes on acetaminophen-induced liver injury

Heldring, M.M.[1], Shaw, A.H.[1] and Beltman, J.B.[1,]*

[1] Division of Drug Discovery and Safety, Leiden Academic Centre for Drug Research, Leiden University, Einsteinweg 55, 2333 CC Leiden, The Netherlands

* Corresponding author, j.b.beltman@lacdr.leidenuniv.nl

List of Contents

Supplementary Figure 1	p. 3
Supplementary Figure 2	p. 4
Supplementary Figure 3	p. 5
Supplementary Figure 4	p. 6
Supplementary Table 1	p. 7
Supplementary Table 2	p. 8
Supplementary Table 3	p. 9
Supplementary Table 4	p. 10
Supplementary Table 5	p. 11
Supplementary Methods	p. 12
Supplementary References	p. 13

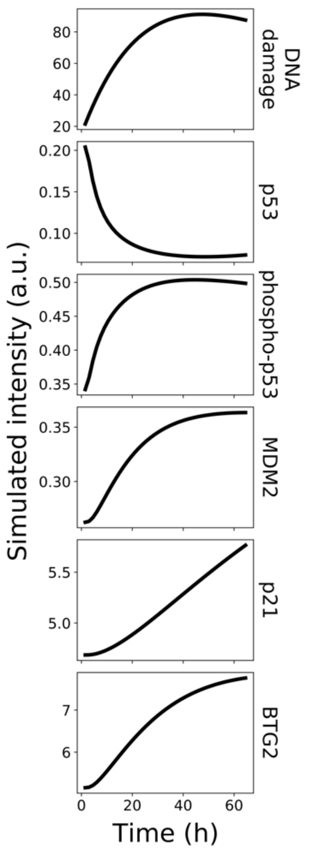

Supplementary Figure 1. Simulation of the adjusted DNA damage response model at 5 µM cisplatin[1].

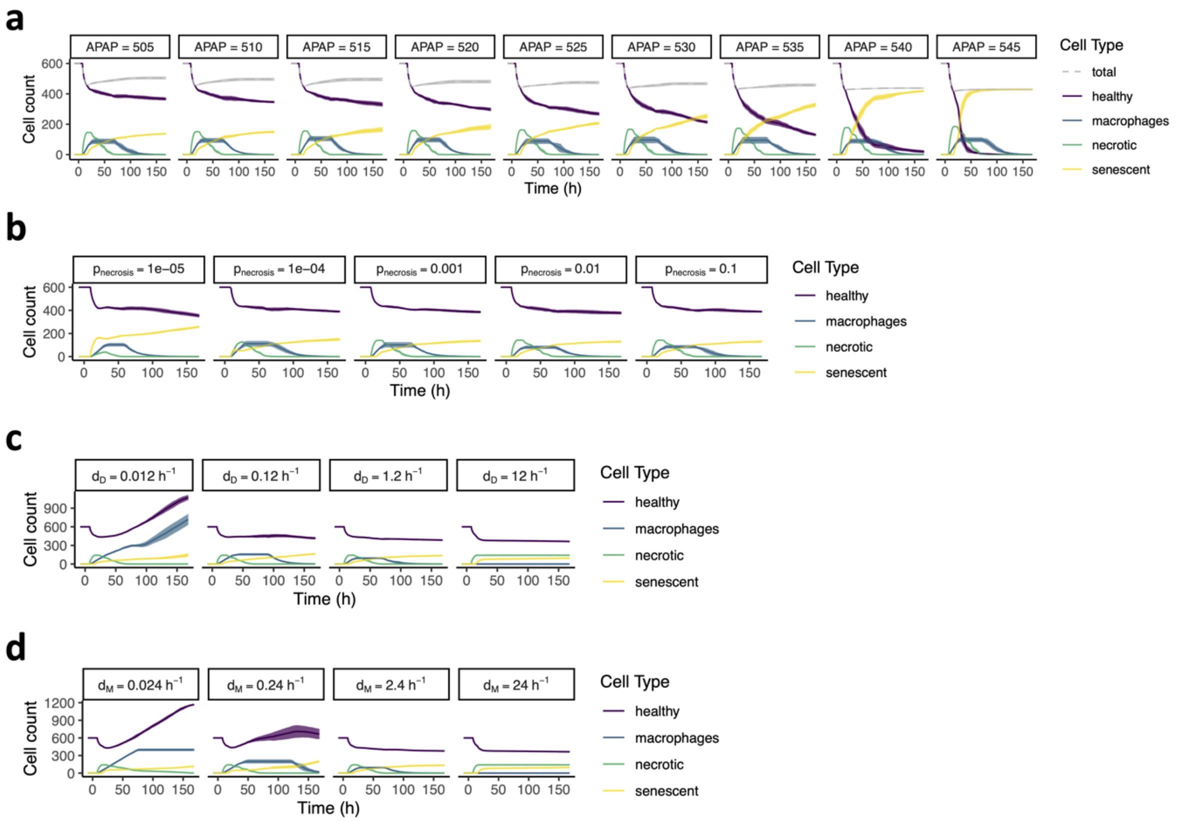

Supplementary Figure 2. Population dynamics of healthy, necrotic, senescent and total number of hepatocytes and the number of macrophages. **(a)** Cell numbers after 505-545 APAP treatment. **(b)** Cell numbers after 500 APAP exposure, with different parameter values for the probability of necrosis, $p_{necrosis}$. **(c-d)** Cell numbers after 500 APAP exposure, with different parameter values for the degradation rates of DAMPs, with d_D = 1.2 h^{-1} the default value, **(c)** or MCP-1, with d_M = 2.4 h^{-1} the default value **(d)**. Results are based on 10 simulations per condition represented as mean ± sd.

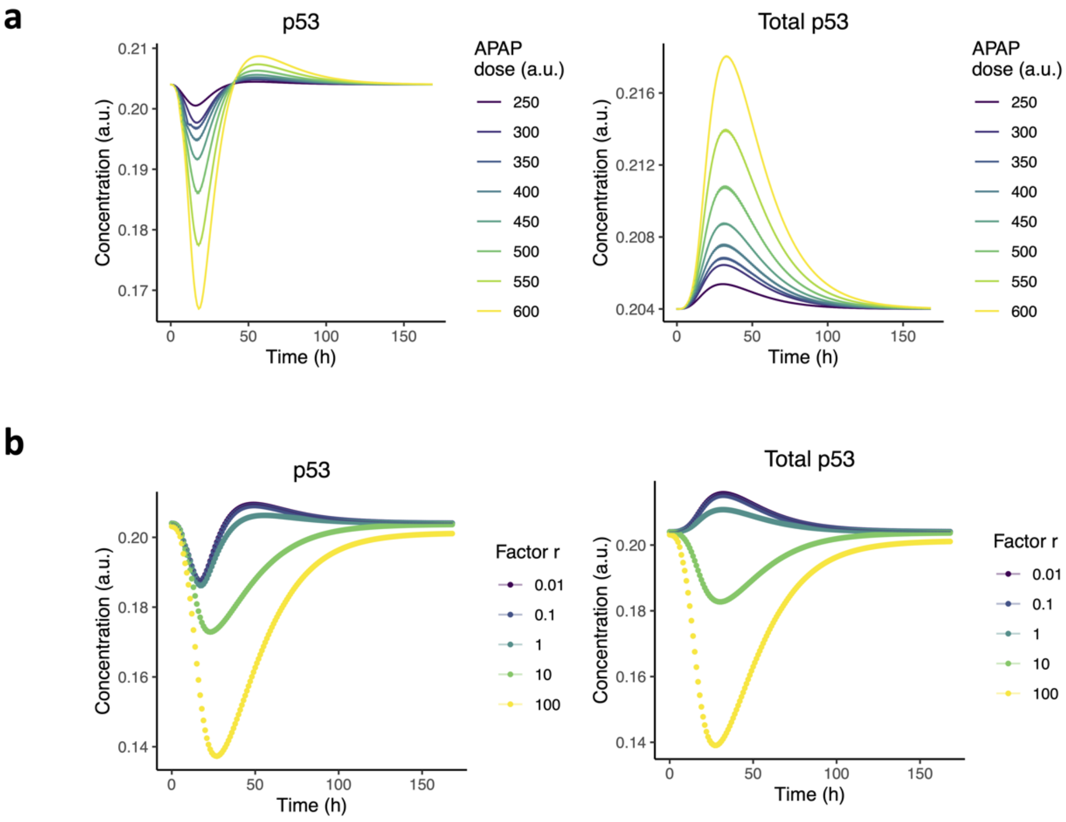

Supplementary Figure 3. Activation of the DNA damage response proteins upon APAP exposure. **a-b)** Population protein expression mean of unphosphorylated p53 (left) and total p53, i.e., the sum of unphosphorylated p53 and phospho-p53 (right), **(a)** at different APAP exposure concentrations or **(b)** under different feedback strengths of MDM2 on p53 after exposure to 500 APAP. Factor r is the multiplication factor used to scale the MDM2 feedback on p53 and phospho-p53 (either up or down). Due to the deterministic character of the intracellular dynamics, there is very little variation between different simulations per condition. Results are based on 10 simulations per condition represented as mean ± sd.

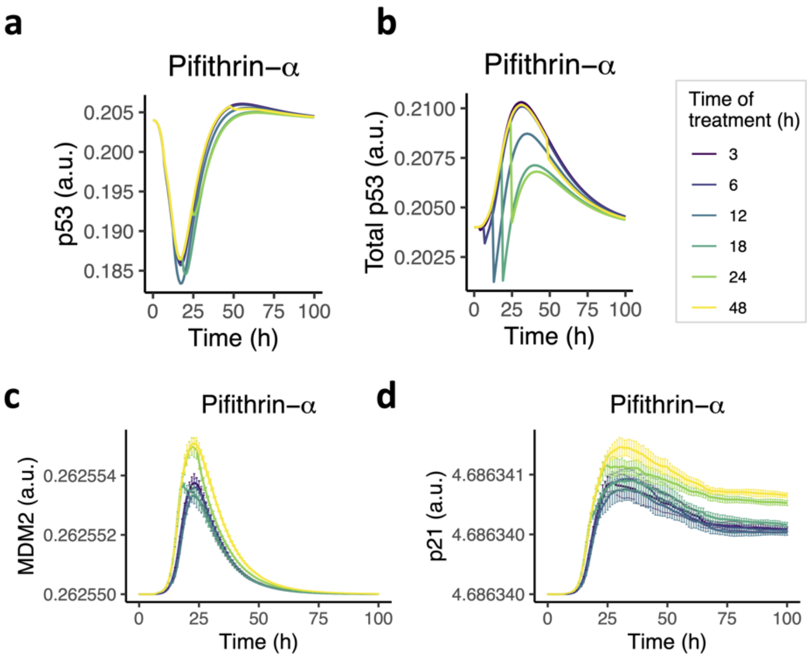

Supplementary Figure 4. Effect of pifithrin-α therapy on p53, MDM2 and p21 expression levels. **a-d)** Population protein expression mean of **(a)** unphosphorylated p53, **(b)** total p53, i.e., the sum of unphosphorylated p53 and phospho-p53, **(c)** MDM2 and **(d)** p21 after *in silico* imitation of pifithrin-α therapy at 3, 6, 12, 18, 24 or 48 hours after exposure to 500 APAP. Results are based on 10 simulations per condition represented as mean ± sd.

Supplementary Table 1. Comparison of administered APAP dose and blood plasma concentration 15-30 minutes after administration

**Organism**	**Mode**	**Dose (mg/kg)**	**Blood plasma concentration (µg/ml)**	**Time (min)**	**Ratio** $\left(\frac{\text{dose}}{\text{concentration}}\right)$	**Reference**
human	oral	20	~ 20	30	~ 1	[2]
human	oral	12	~ 12	30	~ 1	[2]
human	oral	~ 17	~ 16	30	~ 1	[3]
mouse	intraperitoneal	150	106	15	1.4	[4]
mouse	intraperitoneal	300	150	15	2	[5]
mouse	intraperitoneal	350	350	20	1	[6]
mouse	intraperitoneal	375	300	15	1.25	[7]
mouse	intraperitoneal	500	334	15	1.5	[4]
mouse	intraperitoneal	600	425	15	1.4	[5]
mouse	intraperitoneal	700	892	20	0.8	[8]

Supplementary Table 2. Coordinates of the hexagon vertices on a 200x200 lattice

Location	Vertex coordinate x	Vertex coordinate y
Bottom left	50	5
Bottom right	150	5
Mid left	5	100
Mid right	195	100
Top left	50	195
Top right	150	195

Supplementary Table 3. Cellular Potts model (CPM) parameter values

**Parameter**	**Value**	**Description**
$\mathcal{J}_{\tau(\sigma,\sigma')}$	2.2	Surface energy between CPM entities, where $\tau(\sigma) \in \{\text{hepatocyte, Kupffer cell, necrotic cell, macrophage, CV, PV}\}$ and σ the individual cells
$\mathcal{J}_{\tau(\sigma,medium)}$	0.8	Surface energy between CPM entities and medium, where $\tau(\sigma) \in \{\text{hepatocyte, Kupffer cell, necrotic cell, macrophage, CV, PV}\}$ and σ the individual cells
A_{hep}	35	Target area of hepatocytes
A_{kupf}	3.5	Target area of Kupffer cells, assuming $A_{kupf} = 0.1 \cdot A_{hep}$
$A_{m\varphi}$	17.5	Target area of macrophages, assuming $A_{m\varphi} = 0.5 \cdot A_{hep}$
λ_A	1	Strength of the area constraints for hepatocytes, Kupffer cells and macrophages
$\mu_{m\varphi \to M}$	600[a], 2[b], 100[c]	Chemotactic strengths of macrophages in direction of increasing concentration of the MCP-1 gradient, in a) the periportal area, b) the vicinity of necrotic cells and c) elsewhere.

Supplementary Table 4. Parameter values of ODEs that describe APAP metabolism

Para-meter	Value	Unit	Biological interpretation	Derivation	Ref
k_u	0.48	hr^{-1}	APAP uptake rate	-	-
k_e	0.009	hr^{-1}	APAP efflux rate	-	-
d_{GSH}	0.0828	hr^{-1}	GSH degradation rate	2 day^{-1} ~ $\frac{2}{288}$ MCS^{-1} ~ $\frac{2}{288} \cdot 12$ hr^{-1}	9
d_S	0.0833	hr^{-1}	PAPS degradation rate	2 day^{-1} ~ $\frac{2}{288}$ MCS^{-1} ~ $\frac{2}{288} \cdot 12$ hr^{-1}	9
k_g	0.125	hr^{-1}	Glucuronidation rate	2.99 day^{-1} ~ $\frac{2.99}{288}$ MCS^{-1} ~ $\frac{2.99}{288} \cdot 12$ hr^{-1}	9
k_{P450}	0.0131	a.u. hr^{-1}	Oxidation rate	0.315 day^{-1} ~ $\frac{0.315}{288}$ MCS^{-1} ~ $\frac{0.315}{288} \cdot 12$ hr^{-1}	9
k_N	0.0013	hr^{-1}	Reverse oxidation rate	0.0315 day^{-1} ~ $\frac{0.0315}{288}$ MCS^{-1} ~ $\frac{0.0315}{288} \cdot 12$ hr^{-1}	9
b_S	0.833	a.u. hr^{-1}	Basal PAPS synthesis	$\frac{P_{in} \cdot d_S}{10}$ mg L^{-1} hr^{-1}, with P_{in} a reference intracellular APAP concentration of 100 mg L^{-1}	9
k_S	0.0125	a.u. hr^{-1}	Sulfation rate	$\frac{k_g \cdot d_S}{b_S}$	9
k_{GSH}	0.408 – 1.242; cell position dependent	a.u. hr^{-1}	Conjugation rate of NAPQI with GSH	Assuming conjugation with GSH ($k_{GSH} \cdot G$) is 1000 times faster than oxidation by cytochrome P450 ($k_{P450} \cdot P450$), i.e., $1000 \cdot \frac{k_{P450} \cdot P450}{G_0}$, where P450 is the cytochrome P450 concentration	-
b_{GSH}	1.14 – 2.292; cell position dependent	a.u. hr^{-1}	Basal GSH production rate	Assuming steady state at t_0, i.e., $d_{GSH} \cdot G_0$, where G_0 is the initial GSH concentration	-
k_{PSH}	0.12 – 0.17; cell position dependent	hr^{-1}	NAPQI-cys formation rate	$0.01 \cdot k_{GSH} \cdot \frac{b_{GSH}}{d_{GSH}}$ ~ $0.01 \cdot k_{GSH} \cdot G_0$, where G_0 is the initial GSH concentration	9
P_{ex}	500	a.u.	Initial extracellular APAP concentration	-	-
P_{in}	0	a.u.	Initial intracellular APAP concentration	-	-
S_0	10	a.u.	Initial PAPS concentration	Assuming steady state at t_0, i.e., $\frac{b_S}{d_S}$	-
N_0	0	a.u.	Initial NAPQI concentration	-	-
G_0	13.74 – 27.84; cell position dependent	a.u.	Initial GSH concentration	Assuming i) a sigmoidal dependency of GSH concentration on distance from the CV center of mass (scaled between 0 and 1), ii) a two-fold difference between the minimum and maximum GSH concentration [10–13], and iii) a minimum initial GSH concentration of 13.74[(1)], i.e., $1 + \frac{\frac{1}{1+e^{10 \cdot (\text{distance}-0.5)}}}{0.07278}$	9
C_0	0	a.u.	Initial NAPQI-cys concentration	-	-
P450	1 – 1.3; cell position dependent	a.u.	P450 concentration	Assuming i) a sigmoidal dependency of P450 concentration on distance from the CV center of mass (scaled between 0 and 1), ii) a 1.3-fold difference between the minimum and maximum P450 concentration [14,15], and iii) a minimum P450 concentration of 1[(2)], i.e., $3.333 + \frac{\frac{1}{1+e^{10 \cdot (\text{distance}-0.5)}}}{3.333}$	9

(1) The initial APAP concentration P_0 reported by Reddyhoff et al. (2015) is approximately $2 \cdot 10^{15}$ times smaller than P_{in} in our model. With the same ratio, the G_0 concentration of $6.87 \cdot 10^{-15}$ mol $cell^{-1}$ in Reddyhoff et al. (2015) would correspond to 13.74 mg L^{-1}.

(2) P450 species is not consumed in the model of Reddyhoff et al. (2015) and therefore implicitly has value 1. We therefore assumed a minimum P450 concentration of 1.

Supplementary Table 5. Parameter values of ODEs that describe p53 activation and proteins

Parameter	Value	Unit	Biological interpretation	Derivation	Reference
$P53_0$	0.204	au	initial amount of p53	Estimate for the model without p53 mRNA	1
$P53P_0$	0	au	initial amount of phospho-p53	Assuming no initial phospho-p53	-
$MDM2_0$	0.26255	au	initial amount of MDM2	Estimate for the model without p53 mRNA	1
$P21_0$	4.68634	au	initial amount of p21	Estimate for the model without p53 mRNA	1
k_{dp}	0.614547	hr^{-1}	dephosphorylation rate of phospho-p53	Estimate for the model without p53 mRNA. The value used for the spatial model are corrected for change in time units, i.e., 0.614547 $hr^{-1} \sim \frac{0.614547}{18}$ MCS^{-1}	1
kd_{p53p}	0.0003218	hr^{-1}	degradation rate of phospho-p53	Estimate for the model without p53 mRNA and corrected for change in time units, i.e., 0.0003218 $hr^{-1} \sim \frac{0.0003218}{18}$ MCS^{-1}	1
kd_{p53}	0.0711	hr^{-1}	degradation rate of p53	Estimate for the model without p53 mRNA. The value used for the spatial model are corrected for change in time units, i.e., 0.0711 $hr^{-1} \sim \frac{0.0711}{18}$ MCS^{-1}	1
$kd_{p53\ mdm2}$	$3.69 \cdot 10^{-5}$	$au^{-1} \cdot hr^{-1}$	MDM2-dependent degradation rate of p53	Estimate for the model without p53 mRNA. The value used for the spatial model are corrected for change in time units, i.e., $3.69 \cdot 10^{-5}$ $hr^{-1} \sim \frac{3.69 \cdot 10^{-5}}{18}$ MCS^{-1}	1
$kd_{p53p\ mdm2}$	0.10386	$au^{-1} \cdot hr^{-1}$	MDM2-dependent degradation rate of phospho-p53	Estimate for the model without p53 mRNA. The value used for the spatial model are corrected for change in time units, i.e., 0.10386 $hr^{-1} \sim \frac{0.10386}{18}$ MCS^{-1}	1
ks_{mdm2}	0.001803	$au \cdot hr^{-1}$	synthesis rate of MDM2	Assuming steady state at t=0, i.e., $kd_{MDM2} \cdot MDM2_0$	-
$ks_{mdm2\ p53p}$	0.05012	$au \cdot hr^{-1}$	phospho-p53-dependent synthesis rate of MDM2	Estimate for the model without p53 mRNA. The value used for the spatial model are corrected for change in time units, i.e., 0.05012 $hr^{-1} \sim \frac{0.05012}{18}$ MCS^{-1}	1
ks_{p21}	0.001308	$au \cdot hr^{-1}$	synthesis rate of p21	Assuming steady state at t=0, i.e., $kd_{p21} \cdot P21_0$	-
$ks_{p21\ p53p}$	0.25631	$au \cdot hr^{-1}$	phospho-p53-dependent synthesis rate of p21	Estimate for the model without p53 mRNA. The value used for the spatial model are corrected for change in time units, i.e., 0.25631 $hr^{-1} \sim \frac{0.25631}{18}$ MCS^{-1}	1
Km_{mdm2}	0.2962	au	Michaelis-Menten constant for MDM2	Estimate for the model without p53 mRNA	1
Km_{p21}	0.74052	au	Michaelis-Menten constant for p21	Estimate for the model without p53 mRNA	1
k_p	0.0502	hr^{-1}	phosphorylation rate of p53	Estimate for the model without p53 mRNA. The value used for the spatial model are corrected for change in time units, i.e., 0.0502 $hr^{-1} \sim \frac{0.0502}{18}$ MCS^{-1}	1
ks_{p53}	0.0008069	$au \cdot hr^{-1}$	synthesis rate of p53	Assuming steady state at t=0, i.e., $kd_{p53} \cdot P53_0 + kd_{p53\ MDM2} \cdot MDM2_0 \cdot P53_0$	-
kd_{mdm2}	0.123606	hr^{-1}	degradation rate of MDM2	Estimate for the model without p53 mRNA. The value used for the spatial model are corrected for change in time units, i.e., 0.123606 $hr^{-1} \sim \frac{0.123606}{18}$ MCS^{-1}	1
kd_{p21}	0.0050256	hr^{-1}	degradation rate of p21	Estimate for the model without p53 mRNA. The value used for the spatial model are corrected for change in time units, i.e., 0.0050256 $hr^{-1} \sim \frac{0.0050256}{18}$ MCS^{-1}	1

Supplementary Methods

We removed the ODE equations for the mRNA species from our previously published DNA damage model [1]. The remaining ODEs of the reduced DNA damage model that were used for parameter estimation then became:

$$\frac{\mathrm{dDD}}{\mathrm{dt}} = \mathrm{ks_{DD}} - \mathrm{kd_{DD}} \cdot \mathrm{DD} \cdot \mathrm{P53_p} + \mathrm{S}, \qquad \textit{SEq. 1}$$

$$\frac{\mathrm{dP53}}{\mathrm{dt}} = \mathrm{ks_{p53}} + \mathrm{k_{dp}} \cdot \mathrm{P53_p} - \mathrm{k_p} \cdot \mathrm{P53} \cdot \mathrm{DD} - \mathrm{kd_{p53}} \cdot \mathrm{P53} - \mathrm{kd_{p53\,mdm2}} \cdot \mathrm{P53} \cdot \mathrm{MDM2}, \qquad \textit{SEq. 2}$$

$$\frac{\mathrm{dP53_p}}{\mathrm{dt}} = \mathrm{k_p} \cdot \mathrm{P53} \cdot \mathrm{DD} - \mathrm{k_{dp}} \cdot \mathrm{P53_p} - \mathrm{kd_{p53p}} \cdot \mathrm{P53_p} - \mathrm{kd_{p53p\,mdm2}} \cdot \mathrm{P53_p} \cdot \mathrm{MDM2}, \qquad \textit{SEq. 3}$$

$$\frac{\mathrm{dMDM2}}{\mathrm{dt}} = \mathrm{ks_{mdm2}} + \frac{\mathrm{ks_{mdm2\,p53p}} \cdot \mathrm{P53_p}^4}{\mathrm{Km_{mdm2}^4} + \mathrm{P53_p}^4} - \mathrm{kd_{mdm2}} \cdot \mathrm{MDM2}, \qquad \textit{SEq. 4}$$

$$\frac{\mathrm{dP21}}{\mathrm{dt}} = \mathrm{ks_{p21}} + \frac{\mathrm{ks_{p21\,p53p}} \cdot \mathrm{P53_p}^4}{\mathrm{Km_{p21}^4} + \mathrm{P53_p}^4} - \mathrm{kd_{p21}} \cdot \mathrm{P21}, \text{ and} \qquad \textit{SEq. 5}$$

$$\frac{\mathrm{dBTG2}}{\mathrm{dt}} = \mathrm{ks_{btg2}} + \frac{\mathrm{ks_{btg2\,p53p}} \cdot \mathrm{P53_p}^4}{\mathrm{Km_{btg2}^4} + \mathrm{P53_p}^4} - \mathrm{kd_{btg2}} \cdot \mathrm{BTG2}. \qquad \textit{SEq. 6}$$

Supplementary References

1. Heldring, M. M. *et al.* Model-based translation of DNA damage signaling dynamics across cell types. *PLoS Comput. Biol.* **18**, e1010264 (2022).
2. Prescott, L. F. Kinetics and metabolism of paracetamol and phenacetin. *Br. J. Clin. Pharmacol.* **10 Suppl 2**, 291S-298S (1980).
3. Andreasen, P. B. & Hutters, L. Paracetamol (acetaminophen) clearance in patients with cirrhosis of the liver. *Acta Med. Scand. Suppl.* **624**, 99–105 (1979).
4. Ruepp, S. U., Tonge, R. P., Shaw, J., Wallis, N. & Pognan, F. Genomics and proteomics analysis of acetaminophen toxicity in mouse liver. *Toxicol. Sci.* **65**, 135–150 (2002).
5. Shankar, K. *et al.* Type 1 diabetic mice are protected from acetaminophen hepatotoxicity. *Toxicol. Sci.* **73**, 220–234 (2003).
6. Mirochnitchenko, O. *et al.* Acetaminophen Toxicity: OPPOSITE EFFECTS OF TWO FORMS OF GLUTATHIONE PEROXIDASE *. *J. Biol. Chem.* **274**, 10349–10355 (1999).
7. Mitchell, J. R. *et al.* Acetaminophen-induced hepatic necrosis. I. Role of drug metabolism. *J. Pharmacol. Exp. Ther.* **187**, 185–194 (1973).
8. He, M. *et al.* Effects and mechanisms of rifampin on hepatotoxicity of acetaminophen in mice. *Food Chem. Toxicol.* **50**, 3142–3149 (2012).
9. Reddyhoff, D., Ward, J., Williams, D., Regan, S. & Webb, S. Timescale analysis of a mathematical model of acetaminophen metabolism and toxicity. *J. Theor. Biol.* **386**, 132–146 (2015).
10. Chiba, M. & Pang, K. S. Glutathione depletion kinetics with acetaminophen. A simulation study. *Drug Metab. Dispos.* **23**, 622–630 (1995).
11. Anundi, I., Lähteenmäki, T., Rundgren, M., Moldeus, P. & Lindros, K. O. Zonation of acetaminophen metabolism and cytochrome P450 2E1-mediated toxicity studied in isolated periportal and perivenous hepatocytes. *Biochem. Pharmacol.* **45**, 1251–1259 (1993).
12. Smith, M. T., Loveridge, N., Wills, E. D. & Chayen, J. The distribution of glutathione in the rat liver lobule. *Biochem. J* **182**, 103–108 (1979).

13. Väänänen, H. The distribution of cytochrome P-450-mediated drug oxidation and glutathione in periportal and perivenous rat hepatocytes after phenobarbital treatment. *J. Hepatol.* **2**, 174–181 (1986).

14. Baron, J., Redick, J. A. & Guengerich, F. P. An immunohistochemical study on the localization and distributions of phenobarbital- and 3-methylcholanthrene-inducible cytochromes P-450 within the livers of untreated rats. *J. Biol. Chem.* **256**, 5931–5937 (1981).

15. Diaz Ochoa, J. G. *et al.* A multi-scale modeling framework for individualized, spatiotemporal prediction of drug effects and toxicological risk. *Front. Pharmacol.* **3**, 204 (2012).
